# Developing a decision‐making framework for insect pest management: a case study using *Aphis glycines* (Hemiptera: Aphididae)

**DOI:** 10.1002/ps.6093

**Published:** 2020-10-01

**Authors:** Ashley N Dean, Jarad B Niemi, John C Tyndall, Erin W Hodgson, Matthew E O'Neal

**Affiliations:** ^1^ Department of Entomology Iowa State University Ames IA USA; ^2^ Department of Statistics Iowa State University Ames IA USA; ^3^ Department of Natural Resource Ecology and Management Iowa State University Ames IA USA

**Keywords:** economic analysis, integrated pest management, soybean aphid, resistance management, modeling

## Abstract

**BACKGROUND:**

The profitability of farming varies based on factors such as a crop's market value, input costs and occurrence of resistant pests, all capable of altering the value of pest management tactics in an integrated pest management program. We provide a framework for calculating expected yield and expected net revenue of pest management scenarios, using the soybean aphid (*Aphis glycines*) as a case study. Foliar insecticide and host‐plant resistance are effective management tactics for preventing yield loss from soybean aphid outbreaks; however, pyrethroid‐resistant aphid populations pose a management challenge for farmers. We evaluated eight scenarios relevant to soybean aphid management in Iowa with varying probabilities of aphid outbreaks and insecticide‐resistant aphids occurring.

**RESULTS:**

Our equation suggests that insecticide use is profitable when the probability of an aphid outbreak is ≥29%, and soybean production will become more costly with increasing probability of pyrethroid‐resistant aphids. If farmers continue to use pyrethroids, they will not experience financial consequences from pyrethroid‐resistant aphids until the chance of insecticide resistance is 48%. Aphid‐resistant varieties provided consistent yield and offered the highest net revenue under all conditions.

**CONCLUSION:**

This framework can be used for other crop–pest systems to evaluate the profitability of management tactics and investigate how resistance impacts revenue for farmers. Including the cost of resistance in crop budgets can help farmers and agronomic consultants comprehend these impacts and enhance decision‐making to increase revenue and curb resistance development.

## Graphical abstract

We developed a framework to evaluate the profitability of management scenarios across varying probabilities of a pest outbreak occurring and probabilities that the pest is resistant to the management tactic.

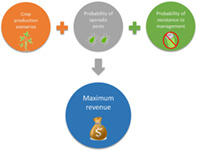

## 1 INTRODUCTION

Financial decision‐making in agriculture employs basic principles to adhere to the financial goals of individual decision‐makers. Agricultural decision‐making is particularly complex, because behavioral differences based on the individual farmer's perception of risk leads to variation in the decision‐making process and differences in farm enterprises often present unique choice options.^1^, ^2^ Management decisions and farmer perception of profitability are further complicated when the severity of a pest infestation is sporadic and unpredictable.^3^ Resistance to management tactics (e.g. insecticide resistance) increases this uncertainty, because failed pest control immediately increases input costs if the farmer seeks additional management (e.g. another insecticide application) and options for future management efforts (e.g. a different mode of action) are typically more expensive. Resistant pest populations decrease crop production per unit of insecticide applied, and increased insecticide use as a result of resistance can result in resurgence of the pest or secondary pest outbreaks.^4^

For insect pests, insecticide use is expected to decrease when resistance is sufficiently prevalent that an alternative management tactic becomes more cost‐effective.^4^ Use of higher priced insecticides can be a consequence of insecticide resistance, which amplifies the cost of resistance if cross‐resistant populations develop and reduces the potential to prevent field‐level resistance.^4^ Financial analyses of pest management options considering contingencies such as resistance, particularly relative to integrated approaches to pest management, can be complex.

Integrated pest management (IPM) seeks to prevent economic damage from pests by coordinating multiple tactics that promote positive biological interactions without negative impacts on productivity.^5^, ^6^, ^7^ Combining pest biology and economics can produce recommendations for selecting among various pest management options.^5^, ^8^ Both theoretical and empirical efforts have been used to develop the economic injury level (EIL) as an indicator of when pest populations reach a level that will produce yield loss above the cost of control. To help prevent this yield loss from occurring, an economic threshold (ET) is set that prompts management action before reaching the EIL.^8^

Greater emphasis on financial evaluations of pest management strategies is needed in the future, especially with increasing occurrence of resistance to management tactics. In systems where crops are economically injured by few pests, such as the Midwest United States, pest management budgets could help farmers comprehend the impact of decision‐making on revenue and guide adoption of IPM principles. Financial models and budgeting techniques for pest management options are most useful if designed to be easily adjusted for different environments or crop–pest systems.^9^ Probability models explicitly address uncertainty associated with decision‐making and allow for inputs to be changed as new information becomes available, which is imperative to the success of a model that accounts for sporadic pests.^10^ Expected financial outcomes can be calculated based on the probability of an event occurring (i.e. a pest outbreak). These outcomes can be used in a prescriptive (i.e. one decision is better than another) or descriptive (i.e. a range of outcomes for a decision) manner for selecting an optimal approach.

The objective of this research was to develop a decision‐making framework to maximize expected yield and revenue in the presence of a pest outbreak. Soybean aphid [*Aphis glycines* Matsumura (Hemiptera: Aphididae)] was used as a case study to test the framework because multiple effective management tactics are available for this sporadic pest. We used Iowa as the basis of a snapshot analysis, both in terms of the pest's status and the inputs required to produce soybeans [*Glycine max* (L.) Merr.]. The pest status of the soybean aphid varies within North America and in other soybean growing regions where the aphid is native.^11^ The goal was to use the framework to reveal how insecticide‐resistant aphids affect revenue and management decisions for farmers and the conditions where aphid‐resistant soybean varieties are most valuable in terms of yield and revenue.

## 2 MATERIALS AND METHODS

### 2.1 Calculating expected yield and expected net revenue

We created equations to calculate yield and net revenue with variation in the probability of a pest outbreak that reduces yield. The effect of management tactics on yield was estimated by considering two probabilities: the probability of a pest outbreak occurring and the probability that the pest is resistant to a management tactic (e.g. insecticide resistance). Expected yield was calculated using Eqn (1):

(1) $$\frac{E\left(Y\right)}{\text{yield}}=p_{n}+p_{o}\times (p_{w}\;I\left[\mathrm{any}\;\text{management}\right]+p_{w}q\;I\left[\mathrm{no}\;\text{management}\right]+p_{s}\;I\left[\text{effective management}\right]+p_{s}q\;I\left[\text{ineffective management}\right]+p_{c}\;I\left[\text{effective management}\right]+p_{c}q\;I\left[\text{ineffective management}\right])$$

where $\frac{E\left(Y\right)}{\text{yield}}$ is the proportion of yield a farmer could expect based on pest incidence, pest characteristics and management decisions. *E*(Y) is the expected yield, calculated by multiplying $\frac{E\left(Y\right)}{\text{yield}}\;$ by yield. Yield is what can be achieved by the crop in the absence of a pest outbreak. The probabilities of no pest outbreak and a pest outbreak occurring are represented by *p*_n_ and *p*_o_, respectively, and sum to 1. An outbreak was defined as a pest population that exceeds an ET and results in yield loss when unmanaged. A pest population susceptible to insecticides is considered the wild‐type, represented as *p*_w_. *I*(A) is an indicator function that is 1 when A is true and 0 when A is false. Management decisions that impact yield when a pest outbreak occurs are represented in the indicator functions (e.g. management when an outbreak occurs does not result in yield loss as long as it is effective, but yield loss is expected when no management occurs or management is ineffective). The proportion of yield expected to be obtained when management decisions result in yield loss is represented by q. In addition to the wild‐type, a pest outbreak can be composed of a population resistant to a single insecticide or an insecticide‐resistant population that also is cross‐resistant to another insecticide, which are represented by *p*_s_ and *p*_c_, respectively. Probabilities for the type of pest population (*p*_w_, *p*_s_ and *p*_c_) sum to 1 and are conditional upon an outbreak occurring.

The effect of management tactics on net revenue was evaluated using a standard calculation for revenue; however, the variables in the equation are considered random in that a farmer does not know their value at the beginning of the season. Therefore, expected net revenue was calculated using Eqn (2):

(2) $$E\left(R\right)=E\left(Y\right)\times E\left(P\right)-E\left(C\right)$$

where *E*(R) is the expected net revenue, *E*(Y) is the expected yield calculated using Eqn (1), *E*(P) is the expected market price of the crop and *E*(C) is the expected variable costs. In deriving this equation, yield and crop price were assumed to be independent of each other for a particular farmer. Because only variable costs were accounted for in our analysis, results depict differences in revenue but do not represent overall profitability of the farming operation.

### 2.2 Decision‐making framework and scenario development

#### 2.2.1 *Decision‐making framework*

**Figure 1:**
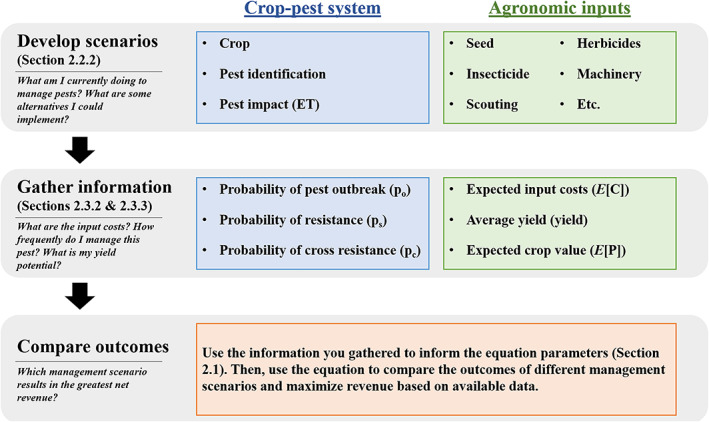
The decision‐making framework for insect pest management involves knowledge of the crop–pest system and agronomic processes. Once pest management scenarios are developed and information gathered, the equations that we developed can be used to compare yield and revenue outcomes for optimal decision‐making.

Managing insect pests requires farmers to make decisions about crop variety, monitoring for pests, and if or when to make insecticide applications. These decisions often are made based on the pest species and perceived risk of an outbreak occurring, and they eventually influence yield and revenue for the farmer. Figure 1 outlines a decision‐making framework for insect pest management, which can be used to compare outcomes from different pest management scenarios based on information gathered for the equations in Section 2.1. We demonstrate how to use this framework with a case study in Section 2.3.

#### 2.2.2 *Scenario development*

Five components of decision‐making relevant to the equation were outlined: the crop variety's susceptibility to the pest, the crop variety's herbicide tolerance trait, insecticide applications, scouting and the pest's susceptibility to the management tactic. This is not an exhaustive list but instead a subset of possible decisions that could be explored. One option from each of these components can be combined to form scenarios for insect pest management. These scenarios are given an abbreviation based on the five components (Table 1).

**Table 1:** Decision‐making outline for scenarios

Scenario	Variety type				Scouting			Spray type			Pests	
	Aphid‐susceptible (S)	Aphid‐resistant (R)	Herbicide‐tolerant (H)	Conventional (C)	None (N)	Efficacy (E)	Monitoring (M)	None (X)	Prophylactic (P)	Threshold‐based (T)	Wild‐type (W)	Insecticide‐resistant (I)
SHXN‐W	●		●		●			●			●	
SHPN‐W	●		●		●				●		●	
SHPE‐W	●		●			●			●		●	
RCXN‐W		●		●	●			●			●	
SHXN‐I	●		●		●			●				●
SHPN‐I	●		●		●				●			●
SHPE‐I	●		●			●			●			●
RCXN‐I		●		●	●			●				●

Black dots indicate a component that is part of the scenario.

The crop variety can be either susceptible (S) or resistant (R) to the pest. The crop variety also can be either herbicide‐tolerant (H) or conventional (C), as annual weed management decisions impact variety selection and input costs. Varieties suitable for organic soybean production [certified by the United States Department of Agriculture (USDA)] also are available but are not considered separately in our framework. Insecticides are either not applied (X), applied prophylactically (P; also referred to as calendar‐based applications) or applied only when an ET is reached (T). Prophylactic applications would not require the farmer to monitor the population in the field (N), but ET‐based applications require monitoring (M) to make a timely insecticide application. Monitoring implies scouting the field during a growing season, but it also can be performed in a more limited manner to determine if the insecticide was effective. We refer to this as efficacy scouting (E), which occurs only following the application of an insecticide to ensure the pest was controlled. The pest could be either susceptible, deemed wild‐type (W), or resistant to insecticides (I), although resistance to another management option could be substituted. To demonstrate how to use these components to form a pest management scenario, SHXN‐W represents a scenario where a susceptible, herbicide‐tolerant variety is planted, no insecticide is applied, no scouting occurs and the pest is susceptible to insecticides (Table 1).

### 2.3 The soybean aphid case study

#### 2.3.1 *Soybean aphid management*

Soybean aphid is an invasive pest of soybean in the United States, first documented in Wisconsin in 2000.^12^ Soybean aphid can reduce pod set, seed size and plant height, resulting in yield losses as great as 50% in the Midwest.^13^, ^14^ Because soybean aphid outbreaks are sporadic, the impact of this pest varies in time and space, posing a challenge for profitable management.^11^, ^15^, ^16^

Insecticides and host‐plant resistance are equally effective management tactics for managing soybean aphid in Iowa, and aphid‐resistant varieties eliminate the need for insecticides when management is required.^14^, ^17^, ^18^, ^19^, ^20^, ^21^, ^22^ Subpopulations of soybean aphids can survive on aphid‐resistant varieties, but these virulent biotypes are at too low a frequency in the Midwest to produce outbreaks on most varieties.^23^, ^24^, ^25^ Pyrethroids and organophosphates (Groups 3A and 1B, respectively^26^) are commonly used classes of insecticides for soybean production, but reports of soybean aphid populations resistant to pyrethroids are a significant concern for farmers in the Midwest.^27^, ^28^

Profitability of host‐plant resistance and foliar‐applied insecticides for soybean aphid management can be compared using the equations in Section 2.1. Because these tactics work equally well to protect yield, it is assumed they would not be combined within a field season for soybean aphid management (e.g. aphid‐resistant varieties would not be treated with insecticide to manage soybean aphid). This may change if virulent biotypes become more frequent with increased use of aphid‐resistant soybean varieties. Therefore, we explored scenarios where these tactics are implemented alone to reflect common management practices in Iowa and scenarios that may become a reality with increasing prevalence of insecticide‐resistant aphids (Table 1).

Eight scenarios are discussed in this manuscript, although more scenarios are possible (detailed in Tables S1 and S2). In the soybean aphid case study, aphid‐susceptible soybean varieties are either herbicide‐tolerant or conventional; however, as of 2019, soybean aphid resistance is only available in conventional (i.e. not herbicide tolerant) soybean varieties.^22^ All soybean varieties were assumed to have the same yield potential, although yield differences can be easily incorporated. To display results as expected yield, *E*(Y), we multiplied $\frac{E\left(Y\right)}{\text{yield}}$, the outcome of Eqn (1), by the ten‐year average yield in Iowa (3537 kg ha^−1^).^29^ Prophylactic applications were assumed to occur as a tank‐mix with fungicide applications at flowering (R1) and prevent yield loss as well as an insecticide applied when the ET is reached.^13^, ^30^, ^31^, ^32^ By incorporating an insecticide resistance parameter in the equation, we can account for reapplication of insecticides when scouting after an application (i.e. efficacy scouting) reveals aphids were not controlled by the insecticide.

In the case study, *p*_s_ refers to pyrethroid‐resistant aphids and *p*_c_ refers to pyrethroid‐resistant aphids that are cross‐resistant to organophosphates. Cross‐resistance is not discussed here (instead, see Tables S1 and S2). For scenarios with insecticide‐resistant aphids, it is assumed that farmers are using the least expensive insecticide (in this system, pyrethroids) if it is still effective, and the next cheapest option is an organophosphate followed by sulfoxaflor. Therefore, pyrethroid‐resistant populations that are cross‐resistant to organophosphates are assumed to arise before resistance to an organophosphate alone. Other insecticides can be easily incorporated for different purposes. Parameter values for each scenario can be found in Tables 2, S1 and S2.

**Table 2:** Parameter values for Eqn (1)

Scenario	Parameters					
	*p*_n_ ^†^	*p*_o_ ^‡^	*q* ^§^	*p*_w_ ^¶^	*p*_s_ ^¶^	*p*_c_ ^¶^
SHXN‐W	0–1	0–1	0.873	1	0	0
SHPN‐W	0–1	0–1	0.873	1	0	0
SHPE‐W	0–1	0–1	0.873	1	0	0
RCXN‐W	0–1	0–1	1	1	0	0
SHXN‐I	0.565	0.435	0.873	0–1	0–1	0
SHPN‐I	0.565	0.435	0.873	0–1	0–1	0
SHPE‐I	0.565	0.435	0.873	0–1	0–1	0
RCXN‐I	0.565	0.435	1	0–1	0–1	0

^†^ For *p*_n_ (the probability of no outbreak), increments of 0.1 are used for scenarios with wild‐type aphids (‐W). Scenarios with insecticide‐resistant aphids (‐I) have a constant value of 0.565, which is the average of 23 site‐years in Iowa.^33^

^‡^ *p*_o_ (the probability of an outbreak) is always the inverse of *p*_n_, as these parameters sum to 1.

^§^ *q* is the proportion of yield expected when an outbreak occurs and management decisions result in yield loss. For aphid‐susceptible varieties, this value is 0.873 (12.7% yield loss). For aphid‐resistant varieties, this value is 1 (0% yield loss).

^¶^ *p*_w_, probability of wild‐type aphids; *p*_s_, probability of insecticide‐resistant aphids; and *p*_c_, probability of insecticide‐resistant aphids that are cross‐resistant to another insecticide group. Scenarios with wild‐type aphids (‐W) have *p*_w_ = 1 and *p*_s_ and *p*_c_ = 0. Scenarios with insecticide‐resistant aphids (‐I) use increments of 0.1 for either *p*_w_, *p*_s_ or *p*_c_, which must sum to 1.

#### 2.3.2 *Frequency of aphid outbreaks and yield loss*

Evaluations of insecticides for efficacy against soybean aphid have been conducted since 2005 in Iowa.^33^ From these evaluations, data were compiled from 12 years and three sites for a total of 23 site‐years: Iowa State University (ISU) Northwest Research Farm (2011–2018), ISU Northeast Research Farm (2007–2018) and ISU Johnson Research Farm (2009–2011). Soybean aphid outbreaks, which were considered to have occurred when populations exceeded the ET before the R6 growth stage,^30^ occurred 43.5% of the time across all site‐years. These locations were selected to evaluate insecticides based on the high likelihood of an aphid outbreak occurring; therefore, this probability does not reflect a state or region‐wide risk of an outbreak. Because aphid population dynamics are difficult to predict,^15^, ^16^ we assumed an outbreak probability of 43.5% for a location with a high risk of an outbreak.

Yield loss was calculated in each site‐year with the equation 100 − [(U ÷ H) × 100], where U is the yield of the untreated control and H is the insecticide treatment with the highest yield; the insecticide treatment with the highest yield was assumed to represent the potential yield for soybean for that site‐year. Overall, mean yield loss in an outbreak year was 12.7% (*q* = 0.873 for aphid‐susceptible varieties). Aphid‐resistant varieties were not expected to experience aphid populations that result in yield loss (*q* = 1) based on previous research.^22^ We assumed that insecticide‐resistant aphids do not cause additional yield loss beyond what a wild‐type population would cause during an outbreak; therefore, yield loss is never >12.7% in any scenario.

#### 2.3.3 *Cost of soybean production in Iowa*

Costs associated with soybean aphid management tactics differ because more inputs are required to manage populations with an insecticide application (e.g. insecticide and application costs), and aphid‐resistant varieties are not yet available in highly desired herbicide‐tolerant backgrounds.^22^ This results in different seed and herbicide costs for aphid‐susceptible and aphid‐resistant soybean varieties in the analysis. Because 94% of soybean in the United States is planted with herbicide‐tolerant varieties, an aphid‐susceptible + herbicide‐tolerant variety was considered the standard practice for soybean production.^34^

The total cost of producing soybean in Iowa, from planting through harvest, was accounted for in all scenarios using a snapshot analysis from 2018 (all costs are in 2018 USD). Typical management practices for soybean production were outlined, and costs were appraised for each practice (Table 3). The 2018 ISU Ag Decision Maker crop budgets and custom rate survey data provided establishment and input costs other than seed and insecticide.^35^, ^36^ Regional online chemical dealers were surveyed to estimate 2018 costs for insecticides, and prices for bulk products were converted to a per hectare cost based on the labelled application rate. Pesticide costs are displayed in Table 4. Baseline seed costs for aphid‐susceptible and aphid‐resistant soybean varieties (Table 5) were estimated by averaging 2018 catalog prices from regional seed dealers. Note that the listed price of the seed was used for the analysis, which does not reflect potential discounts farmers may receive for bulk, cash, or advance purchases. An equal seeding rate of 345 940 seeds ha^–1^ was assumed for each variety (industry standard of 140 000 seeds bag^–1^; assumed one bag acre^–1^).

**Table 3:** Management steps for soybean production and costs (2018 figures)

Management step^†^	Cost (US$ ha^–1^)	Source (citation)
Tillage	39.70	ISU Custom Rate Survey ^31^
Total herbicide product	Variable	ISU crop budgets ^30^
Total herbicide application	43.61	ISU Custom Rate Survey ^31^
Planting cost	73.39	ISU Custom Rate Survey ^31^
Seed cost	Variable	Regional survey; see Table 5
Early season scouting	10.50 per activity^‡^	ISU Custom Rate Survey ^31^
Insecticide cost	Variable	Pesticide survey; see Table 4
Insecticide application	20.02 per application^§^	ISU Custom Rate Survey ^31^
In‐season scouting	10.50 per activity^‡^	ISU Custom Rate Survey ^31^
Harvest	120.83	ISU Custom Rate Survey ^31^
Land rental	548.56	Cash rental rate survey ^33^

^†^ Management steps may not fully represent an individual's operation but were considered standard steps for soybean production in Iowa.

^‡^ It was assumed that scouting would be done twice early in the season for weeds, stand issues and seedling diseases. Efficacy scouting occurred once or twice in our analysis, depending on whether a second application was needed, and monitoring required scouting six times throughout the season.

^§^ A maximum of two insecticide applications occurred, depending on the scenario.

**Table 4:** Pesticide costs (2018 figures)

Pesticide type	Cost (US$ ha^–1^)	*N* ^§^
Herbicides^†^	104.16	2
Conventional	118.61	1
Herbicide‐tolerant	89.70	1
Insecticides^‡^	22.88	8
Group 1B	21.98	3
Group 3A	13.83	4
Group 4C	61.78	1

^†^ The costs for ‘conventional’ and ‘herbicide‐tolerant’ depict the average cost of herbicide products that are applied to conventional soybean varieties or herbicide‐tolerant soybean varieties, respectively.^35^

^‡^ Group 1B refers to organophosphate insecticides, Group 3A refers to pyrethroid insecticides, and Group 4B refers to sulfoxaflor insecticides.^26^

^§^ The sample size used to compute the mean in column 2.

**Table 5:** Regional soybean seed costs (2018 figures)

Aphid trait^†^	Herbicide trait^‡^	Mean cost (US$ ha^–1^)^§^	*N* ^¶^
Aphid‐susceptible		135.62	34
	None	116.11	17
	Conventional	117.65	11
	Organic	113.28	6
	Herbicide‐tolerant	**155.13**	17
	Roundup Ready®	152.43	6
	Roundup Ready® Xtend	167.06	6
	LibertyLink®	144.06	5
Aphid‐resistant		**111.56**	11
*Rag1*	Organic	103.78	4
*Rag1 + Rag2*	Conventional	114.37	5
	Organic	120.10	2

Bolded costs indicate means used in revenue calculations.

^†^ Soybean varieties were either susceptible or resistant to soybean aphid. Host‐plant resistant varieties contained either the *Rag1* gene or the *Rag1 + Rag2* gene pyramid that confer resistance to the soybean aphid.

^‡^ Conventional, not herbicide‐tolerant but not organic; Organic, USDA certified organic seed; Roundup Ready®, tolerant to glyphosate herbicides; Roundup Ready® Xtend, tolerant to glyphosate + dicamba herbicides; LibertyLink®, tolerant to glufosinate herbicides.

^§^ All seed prices were converted to the 140 000 seeds bag^–1^ industry standard. 345 940 seeds ha^–1^ was assumed to be the planting rate.

^¶^ The sample size used to compute the mean in column 3.

Finally, because 53% of Iowa farmland is rented, primarily using cash rental agreements, the 2018 state average land rent was included in the total cost of soybean production.^37^, ^38^ Costs that varied among scenarios included herbicide, soybean seed, scouting activities, insecticide and insecticide application, and all other costs for soybean production were considered fixed. The 2018 calendar year market price of soybean in Iowa (US$0.33 kg^–1^) was used in our calculation of expected net revenue.^39^ We compared each scenario to determine which decisions resulted in the greatest expected net revenue.

#### 2.3.4 *Sensitivity analyses*

Analyses were performed to evaluate how sensitive expected net revenue is to changes in yield, soybean price, aphid‐resistant seed cost and overall input costs because these factors often influence management decisions. If the results are sensitive to these variables, optimal decision‐making could depend on these conditions each year, which are often unpredictable. All factors except the one of interest were held constant in each analysis. These data are not shown but can be recreated using the excel file in the online supporting information.

In order to understand how yield might affect the results of the case study, the ten‐year (2009–2018) state averages for the low (3026 kg ha^−1^) and high (4035 kg ha^−1^) yields in Iowa were used: the original assessment accounted for the ten‐year average yield (3537 kg ha^−1^).^29^ The effect of soybean price was investigated using ten‐year high (2009–2018; US$0.52 kg^–1^) and ten‐year average (US$0.40 kg^–1^) soybean prices: the original assessment accounted for the ten‐year low soybean price (US$0.33 kg^–1^).^39^ To examine how the results would be impacted if current aphid‐resistant varieties were available at a premium cost, it was assumed they would be marketed like herbicide‐tolerant varieties, which have a technology fee. The costs of herbicide‐tolerant and conventional aphid‐susceptible soybean varieties (Table 5) were compared, assuming that the only difference in cost is the fee for herbicide tolerance, and the difference was applied to the cost of conventional aphid‐resistant varieties. Finally, all input costs from the original analysis were reduced by 12%, which reflects the highest decrease in total input costs for herbicide‐tolerant soybean production during the ten‐year period from 2009 to 2018, to investigate the impact of lower input costs.^40^

## 3 RESULTS

### 3.1 Probability of an aphid outbreak occurring – wild‐type aphids

#### 3.1.1 *Expected yield and expected net revenue*

**Figure 2:**
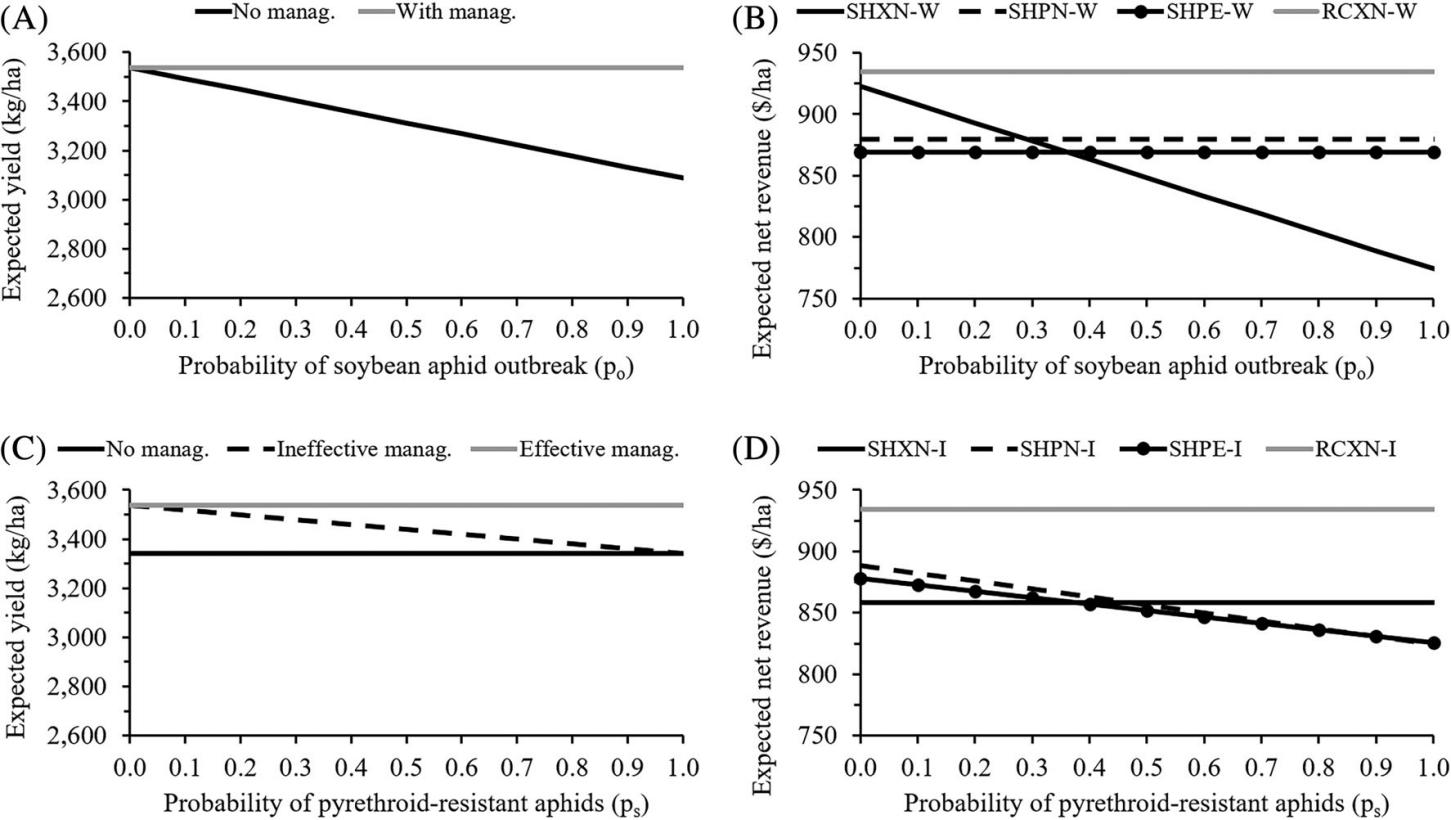
Expected yield (A, C) and expected net revenue (B, D) outcomes for different soybean aphid management scenarios in Iowa. A and B represent results for a range of aphid outbreak probabilities when only wild‐type (insecticide‐susceptible) aphids were present. C and D represent results for a range of probabilities of pyrethroid‐resistant aphids given the probability of an outbreak occurring in high‐risk areas of Iowa (43.5%).

The *E*(Y) was calculated over a range of aphid outbreak probabilities (*p*_o_) for scenarios that assumed aphids were susceptible to insecticides (*p*_w_; Table 1). Two outcomes were observed for expected yield when only wild‐type aphids were present. Without management (SHXN‐W), expected yield declined as the probability of an outbreak increased, with a maximum yield loss of 12.7% which occurred when the probability of an outbreak was equal to one [Fig. 2(A)]. When management occurred [Fig. 2(A)], either with an aphid‐resistant soybean variety (RCXN‐W) or insecticide application (SHPN‐W, SHPE‐W), expected yield remained constant over the range of aphid outbreak probabilities.

The *E*(R) decreased as the probability of an outbreak increased without management to protect yield [SHXN‐W; Fig. 2(B)]. All other scenarios had constant, but different, expected net revenue across the range of aphid outbreak probabilities. The greatest expected net revenue occurred with aphid‐resistant varieties (RCXN‐W; US$934.37 ha^–1^). Insecticide application (SHPN‐W, SHPE‐W) offered increased expected net revenue compared to no management (SHXN‐W) when the probability of an outbreak was ≥29%.

#### 3.1.2 *Sensitivity analysis*

When the price of aphid‐resistant varieties was changed to reflect a technology fee, aphid‐resistant varieties always offered increased expected net revenue compared to insecticides. Aphid‐resistant varieties only offered increased expected net revenue compared to no management when the probability of an outbreak was >18%. A higher probability of an outbreak was required for insecticide to offer greater expected net revenue compared to no management when yield was reduced, whereas the opposite was true with higher yields and soybean prices and when input costs were reduced by 12%.

### 3.2 Probability of pyrethroid‐resistant aphids during an outbreak

#### 3.2.1 *Expected yield and expected net revenue*

We held the probability of an outbreak constant (*p*_o_ = 0.435, representing a high‐risk location in Iowa) to estimate the expected yield for a range of probabilities that an aphid outbreak contained pyrethroid‐resistant aphids (*p*_s;_ Table 1). Three outcomes were observed for expected yield when pyrethroid‐resistant aphids were present. Without management (SHXN‐I), expected yield remained constant, regardless of the probability of pyrethroid‐resistant aphids, and was lowered [Fig. 2(C)], reflecting the probability of an outbreak. When a pyrethroid was applied (SHPN‐I), expected yield decreased with increasing probability of pyrethroid‐resistant aphids if no other insecticide was applied to manage the resistant population [Fig. 2(C)]. However, expected yield was unchanged when effective management occurred [Fig. 2(C)]. Effective management could be provided with aphid‐resistant varieties (RCXN‐I) or an insecticide to which the aphids are still susceptible. We assumed that a pyrethroid was applied first, followed by an organophosphate when it failed (SHPE‐I); however, effective management could also result from an initial application of an insecticide other than a pyrethroid (see Tables S1 and S2 for examples).

Without management [SHXN‐I; Fig. 2(D)], expected net revenue remained constant as the probability of pyrethroid‐resistant aphids increased, because we assumed a constant probability of an aphid outbreak occurring (*p*_o_ = 0.435) that resulted in yield loss regardless of the probability of pyrethroid‐resistant aphids and that pyrethroid‐resistant aphids do not impose additional yield loss when unmanaged. Aphid‐resistant varieties (RCXN‐I) offered the greatest expected net revenue (US$934.37 ha^–1^) overall, which remained constant regardless of the probability of pyrethroid‐resistant aphids. An insecticide application decreased expected net revenue with increasing probability of pyrethroid‐resistant aphids (SHPN‐I, SHPE‐I). A single application of a pyrethroid (SHPN‐I) resulted in less expected net revenue than no management (SHXN‐I) once the probability of pyrethroid‐resistant aphids reached 48%. A second application with an organophosphate (SHPE‐I) provided slightly greater expected net revenue than a single application of a pyrethroid only when the probability of pyrethroid‐resistant aphids was >88%.

#### 3.2.2 *Sensitivity analysis*

With lower yield, a second application resulted in less expected net revenue than a single application that failed to protect yield regardless of the probability of resistant aphids. With high soybean prices, a second insecticide application always resulted in increased expected net revenue compared to no management. At average soybean prices, a single and second insecticide application only offered increased expected net revenue compared to no management when the probability of pyrethroid‐resistant aphids was <57% and 63%, respectively. A higher probability of pyrethroid‐resistant aphids was required for an insecticide application to offer greater expected net revenue than no management when input costs were reduced by 12%.

## 4 DISCUSSION

This research contributes to a small number of entomological studies that have explored the economic impacts of decisions made to manage pests.^1^ Only 1% of entomology articles published since 1972 include a financial analysis, even though economic analyses of pest management tactics are critical for adoption.^1^ Our immediate goal was to provide a framework to maximize yield and revenue based on farmer management decisions. We tested our equations using soybean aphid as a case study to compare expected yields and financial outcomes of soybean aphid management tactics in Iowa. We developed scenarios based on the perceived current management practices for soybean aphid; this is not an exhaustive list of all possible scenarios, and a single scenario may not fully represent an individual's farming operation. We used publicly available resources to obtain input costs, yield information and market prices from 2018 for this analysis, which can be updated annually as new data are published. Additionally, information regarding the frequency of soybean aphid outbreaks and associated yield loss can be updated or changed to reflect farm‐level data, and adjustments can be made as data about insecticide‐resistant soybean aphids become available.

When aphids were susceptible to insecticides (i.e. wild‐type), insecticide applications increased net revenue compared to no management when the probability of an outbreak was >29%. Based on data for high‐risk locations in Iowa (i.e. 43.5% chance of an aphid outbreak), insecticide applications are likely to pay off for farmers in the absence of insecticide‐resistant aphids. These results are consistent with empirical studies that prophylactic applications of insecticide for soybean aphid management paid off 19–74% of the time.^31^, ^41^ Threshold‐based insecticide applications are an important component of IPM programs and can be more cost‐effective than prophylactic insecticide applications.^31^ Our investigation of costs associated with threshold‐based insecticide applications (see Tables S1 and S2) suggests that the cost of this approach is high. Information regarding the frequency and cost of scouting fields can enhance the utility of our equations for IPM programs.

Soybean aphid‐resistant varieties always resulted in increased net revenue compared to an insecticide application with our equation, even without a difference in expected yield. The addition of a technology fee would result in decreased net revenue compared to no management, but only when the probability of an outbreak is <18%. The probability of an outbreak occurring in high‐risk areas within Iowa is nearly triple that, indicating that aphid‐resistant varieties are expected to increase revenue for farmers. Pyramided (*Rag1 + Rag2*) aphid‐resistant varieties are available at the same price as comparable aphid‐susceptible varieties and offer the same yield protection as an insecticide application without additional input costs.^14^, ^22^

The financial benefit of aphid‐resistant soybean varieties was even more pronounced when we considered the probability that an aphid outbreak contained pyrethroid‐resistant aphids. Expected net revenue of pyrethroid applications, the cheapest option for farmers, decreased as the probability of pyrethroid resistance increased. When the chance for pyrethroid resistance was >48%, a single pyrethroid application offered less revenue than no management. There was minimal financial benefit of a second application with an organophosphate to manage resistant aphids. Our sensitivity analysis indicated that with lower yields (3026 kg ha^−1^) farmers that expect to experience a field failure of a pyrethroid due to resistant aphids would not recover the cost of an additional insecticide application and would make more money accepting a yield loss rather than protecting yield with a second insecticide.

We excluded the impact and cost of seed‐applied insecticides as a management tactic owing to substantial empirical evidence revealing that seed‐applied insecticides alone do not prevent yield loss from a soybean aphid outbreak and do not maximize revenue.^14^, ^31^, ^42^, ^43^ Our equation also did not account for resurgence of soybean aphid following an insecticide application. Resurgence occurs following an insecticide application when natural enemies that suppress pest populations are decimated. The pest recolonizes the enemy‐free space and can exceed the pre‐insecticide population. The insecticide efficacy evaluations conducted in Iowa included post‐application assessments of soybean aphid populations, and resurgence has not been observed in these studies.^33^ The probability of resurgence could be included in the future, although empirical studies would be required to measure the frequency at which resurgence occurs and the associated yield loss.

We also assumed that populations of virulent biotypes did not establish on aphid‐resistant soybean at densities sufficient to reduce yield.^23^ Since their introduction nearly a decade ago, aphid‐resistant varieties have not been widely adopted by farmers in the United States. Nevertheless, soybean aphid biotypes exist, although their geographical distribution is variable.^24^, ^25^ If farmers experience issues with soybean aphid and switch to host‐plant resistance as a management tactic, we may see an increase in the prevalence of biotypes. However, the rate at which virulence increases in the population is unknown. The aphid‐susceptible varieties that make up the majority of soybeans grown in the United States provide a natural refuge that generate avirulent aphids. Virulent and avirulent biotypes can co‐occur on resistant varieties, producing ‘refuge‐in‐a‐plant’, which would further reduce the frequency of virulent biotypes if a resistance management plan were followed.^44^

We aimed to demonstrate the utility of our framework for estimating yield and net revenue when a pest is resistant to a management tactic: this does not necessarily have to be soybean aphid resistance to insecticides. The resistance and yield loss parameters in the equation could be modified to represent resistance to host‐plant resistant varieties (i.e. biotypes) if that were the user's concern.

## 5 CONCLUSIONS

Given the results of our case study, it is unlikely that farmers in Iowa are experiencing negative consequences from control failures due to pyrethroid‐resistant aphids because noticeable impacts on net revenue were not expected until the probability of pyrethroid‐resistant aphids exceeded 48%. If this is accurate, farmers could be unknowingly contributing to pyrethroid resistance. Our initial analysis suggests that changes to current inputs and management are necessary to maximize revenue for farmers managing soybean aphid in the future as the risk of pyrethroid‐resistant aphids increases. Information regarding the frequency of insecticide‐resistant aphids in the United States, their impact on yield, and their interactions with biotypes and host‐plant resistance would be imperative to enhance decision making with this framework.

Budgets are available for a variety of crops through university extension allowing farmers to estimate field revenue based on yield and expected input costs, but these budgets do not include the financial implications of managing resistant pests.^4^ The framework presented here can have utility as a decision‐making tool where farmers or other decision‐makers can input their operation's costs, yield, and crop price to determine the most profitable management decisions. This framework may be applied to other crop–pest systems or other states where soybean aphid management is a concern, or expanded as part of a larger, more holistic model that accounts for multiple pest species.

## Supporting information


**Appendix S1.** Supporting Information

**Supporting Table S1.** Expected yield (E[Y]) parameters and estimates for all soybean aphid management scenarios analyzed.

**Supporting Table S2.** Expected net revenue (*E*[R]) parameters and estimates for all soybean aphid management scenarios analyzed.
