# Supplementary material for: Developing a decision‐making framework for insect pest management: a case study using Aphis glycines (Hemiptera: Aphididae)
Source: Pest Manag Sci. 2020 Oct 1;77(2):886–94. doi: 10.1002/ps.6093 (PMC7821323; doi:10.1002/ps.6093)
Supplement: Supplementary file 2 — Supporting Table S1. Expected yield (E[Y]) parameters and estimates for all soybean aphid management scenarios analyzed. [file PS-77-886-s002.docx]

Supporting Table S1. Expected yield (*E*[Y]) parameters and estimates for all soybean aphid management scenarios analyzed.

| **Scenario^[[1]](#footnote-1)^** | **Yield (kg/ha)^[[2]](#footnote-2)^** | **q^[[3]](#footnote-3)^** | **p_o_^[[4]](#footnote-4)^** | **p_w_^4^** | **p_s_^4^** | **p_c_^4^** | **^n^ANY^[[5]](#footnote-5)^** | **^n^PY^5^** | **^n^OP^5^** | **^n^SF^5^** | **E(Y) (kg/ha)^[[6]](#footnote-6)^** |
| --- | --- | --- | --- | --- | --- | --- | --- | --- | --- | --- | --- |
| SHXN-W | 3,537.39 | 0.873 | 0 | 1 | 0 | 0 | 0 | 0 | 0 | 0 | 3,537.4 |
|  | 3,537.39 | 0.873 | 0.1 | 1 | 0 | 0 | 0 | 0 | 0 | 0 | 3,492.5 |
|  | 3,537.39 | 0.873 | 0.2 | 1 | 0 | 0 | 0 | 0 | 0 | 0 | 3,447.5 |
|  | 3,537.39 | 0.873 | 0.3 | 1 | 0 | 0 | 0 | 0 | 0 | 0 | 3,402.6 |
|  | 3,537.39 | 0.873 | 0.4 | 1 | 0 | 0 | 0 | 0 | 0 | 0 | 3,357.7 |
|  | 3,537.39 | 0.873 | 0.5 | 1 | 0 | 0 | 0 | 0 | 0 | 0 | 3,312.8 |
|  | 3,537.39 | 0.873 | 0.6 | 1 | 0 | 0 | 0 | 0 | 0 | 0 | 3,267.8 |
|  | 3,537.39 | 0.873 | 0.7 | 1 | 0 | 0 | 0 | 0 | 0 | 0 | 3,222.9 |
|  | 3,537.39 | 0.873 | 0.8 | 1 | 0 | 0 | 0 | 0 | 0 | 0 | 3,178.0 |
|  | 3,537.39 | 0.873 | 0.9 | 1 | 0 | 0 | 0 | 0 | 0 | 0 | 3,133.1 |
|  | 3,537.39 | 0.873 | 1 | 1 | 0 | 0 | 0 | 0 | 0 | 0 | 3,088.1 |
| SHPN-W | 3,537.39 | 0.873 | 0 | 1 | 0 | 0 | 1 | 0 | 0 | 0 | 3,537.4 |
|  | 3,537.39 | 0.873 | 0.1 | 1 | 0 | 0 | 1 | 0 | 0 | 0 | 3,537.4 |
|  | 3,537.39 | 0.873 | 0.2 | 1 | 0 | 0 | 1 | 0 | 0 | 0 | 3,537.4 |
|  | 3,537.39 | 0.873 | 0.3 | 1 | 0 | 0 | 1 | 0 | 0 | 0 | 3,537.4 |
|  | 3,537.39 | 0.873 | 0.4 | 1 | 0 | 0 | 1 | 0 | 0 | 0 | 3,537.4 |
|  | 3,537.39 | 0.873 | 0.5 | 1 | 0 | 0 | 1 | 0 | 0 | 0 | 3,537.4 |
|  | 3,537.39 | 0.873 | 0.6 | 1 | 0 | 0 | 1 | 0 | 0 | 0 | 3,537.4 |
|  | 3,537.39 | 0.873 | 0.7 | 1 | 0 | 0 | 1 | 0 | 0 | 0 | 3,537.4 |

Supporting Table S1. (continued)

| **Scenario** | **Yield (kg/ha)** | **q** | **p_o_** | **p_w_** | **p_s_** | **p_c_** | **^n^ANY** | **^n^PY** | **^n^OP** | **^n^SF** | **E(Y) (kg/ha)** |
| --- | --- | --- | --- | --- | --- | --- | --- | --- | --- | --- | --- |
|  | 3,537.39 | 0.873 | 0.8 | 1 | 0 | 0 | 1 | 0 | 0 | 0 | 3,537.39 |
|  | 3,537.39 | 0.873 | 0.9 | 1 | 0 | 0 | 1 | 0 | 0 | 0 | 3,537.39 |
|  | 3,537.39 | 0.873 | 1 | 1 | 0 | 0 | 1 | 0 | 0 | 0 | 3,537.39 |
| SHPE-W | 3,537.39 | 0.873 | 0 | 1 | 0 | 0 | 1 | 0 | 0 | 0 | 3,537.39 |
|  | 3,537.39 | 0.873 | 0.1 | 1 | 0 | 0 | 1 | 0 | 0 | 0 | 3,537.39 |
|  | 3,537.39 | 0.873 | 0.2 | 1 | 0 | 0 | 1 | 0 | 0 | 0 | 3,537.39 |
|  | 3,537.39 | 0.873 | 0.3 | 1 | 0 | 0 | 1 | 0 | 0 | 0 | 3,537.39 |
|  | 3,537.39 | 0.873 | 0.4 | 1 | 0 | 0 | 1 | 0 | 0 | 0 | 3,537.39 |
|  | 3,537.39 | 0.873 | 0.5 | 1 | 0 | 0 | 1 | 0 | 0 | 0 | 3,537.39 |
|  | 3,537.39 | 0.873 | 0.6 | 1 | 0 | 0 | 1 | 0 | 0 | 0 | 3,537.39 |
|  | 3,537.39 | 0.873 | 0.7 | 1 | 0 | 0 | 1 | 0 | 0 | 0 | 3,537.39 |
|  | 3,537.39 | 0.873 | 0.8 | 1 | 0 | 0 | 1 | 0 | 0 | 0 | 3,537.39 |
|  | 3,537.39 | 0.873 | 0.9 | 1 | 0 | 0 | 1 | 0 | 0 | 0 | 3,537.39 |
|  | 3,537.39 | 0.873 | 1 | 1 | 0 | 0 | 1 | 0 | 0 | 0 | 3,537.39 |
| SHTM-W | 3,537.39 | 0.873 | 0 | 1 | 0 | 0 | 1 | 0 | 0 | 0 | 3,537.4 |
|  | 3,537.39 | 0.873 | 0.1 | 1 | 0 | 0 | 1 | 0 | 0 | 0 | 3,537.4 |
|  | 3,537.39 | 0.873 | 0.2 | 1 | 0 | 0 | 1 | 0 | 0 | 0 | 3,537.4 |
|  | 3,537.39 | 0.873 | 0.3 | 1 | 0 | 0 | 1 | 0 | 0 | 0 | 3,537.4 |
|  | 3,537.39 | 0.873 | 0.4 | 1 | 0 | 0 | 1 | 0 | 0 | 0 | 3,537.4 |
|  | 3,537.39 | 0.873 | 0.5 | 1 | 0 | 0 | 1 | 0 | 0 | 0 | 3,537.4 |
|  | 3,537.39 | 0.873 | 0.6 | 1 | 0 | 0 | 1 | 0 | 0 | 0 | 3,537.4 |
|  | 3,537.39 | 0.873 | 0.7 | 1 | 0 | 0 | 1 | 0 | 0 | 0 | 3,537.4 |
|  | 3,537.39 | 0.873 | 0.8 | 1 | 0 | 0 | 1 | 0 | 0 | 0 | 3,537.4 |
|  | 3,537.39 | 0.873 | 0.9 | 1 | 0 | 0 | 1 | 0 | 0 | 0 | 3,537.4 |
|  | 3,537.39 | 0.873 | 1 | 1 | 0 | 0 | 1 | 0 | 0 | 0 | 3,537.4 |
| SCXN-W | 3,537.39 | 0.873 | 0 | 1 | 0 | 0 | 0 | 0 | 0 | 0 | 3,537.4 |
|  | 3,537.39 | 0.873 | 0.1 | 1 | 0 | 0 | 0 | 0 | 0 | 0 | 3,492.5 |

Supporting Table S1. (continued)

| **Scenario** | **Yield (kg/ha)** | **q** | **p_o_** | **p_w_** | **p_s_** | **p_c_** | **^n^ANY** | **^n^PY** | **^n^OP** | **^n^SF** | **E(Y) (kg/ha)** |
| --- | --- | --- | --- | --- | --- | --- | --- | --- | --- | --- | --- |
|  | 3,537.39 | 0.873 | 0.2 | 1 | 0 | 0 | 0 | 0 | 0 | 0 | 3,447.5 |
|  | 3,537.39 | 0.873 | 0.3 | 1 | 0 | 0 | 0 | 0 | 0 | 0 | 3,402.6 |
|  | 3,537.39 | 0.873 | 0.4 | 1 | 0 | 0 | 0 | 0 | 0 | 0 | 3,357.7 |
|  | 3,537.39 | 0.873 | 0.5 | 1 | 0 | 0 | 0 | 0 | 0 | 0 | 3,312.8 |
|  | 3,537.39 | 0.873 | 0.6 | 1 | 0 | 0 | 0 | 0 | 0 | 0 | 3,267.8 |
|  | 3,537.39 | 0.873 | 0.7 | 1 | 0 | 0 | 0 | 0 | 0 | 0 | 3,222.9 |
|  | 3,537.39 | 0.873 | 0.8 | 1 | 0 | 0 | 0 | 0 | 0 | 0 | 3,178.0 |
|  | 3,537.39 | 0.873 | 0.9 | 1 | 0 | 0 | 0 | 0 | 0 | 0 | 3,133.1 |
|  | 3,537.39 | 0.873 | 1 | 1 | 0 | 0 | 0 | 0 | 0 | 0 | 3,088.1 |
| SCPN-W | 3,537.39 | 0.873 | 0 | 1 | 0 | 0 | 1 | 0 | 0 | 0 | 3,537.4 |
|  | 3,537.39 | 0.873 | 0.1 | 1 | 0 | 0 | 1 | 0 | 0 | 0 | 3,537.4 |
|  | 3,537.39 | 0.873 | 0.2 | 1 | 0 | 0 | 1 | 0 | 0 | 0 | 3,537.4 |
|  | 3,537.39 | 0.873 | 0.3 | 1 | 0 | 0 | 1 | 0 | 0 | 0 | 3,537.4 |
|  | 3,537.39 | 0.873 | 0.4 | 1 | 0 | 0 | 1 | 0 | 0 | 0 | 3,537.4 |
|  | 3,537.39 | 0.873 | 0.5 | 1 | 0 | 0 | 1 | 0 | 0 | 0 | 3,537.4 |
|  | 3,537.39 | 0.873 | 0.6 | 1 | 0 | 0 | 1 | 0 | 0 | 0 | 3,537.4 |
|  | 3,537.39 | 0.873 | 0.7 | 1 | 0 | 0 | 1 | 0 | 0 | 0 | 3,537.4 |
|  | 3,537.39 | 0.873 | 0.8 | 1 | 0 | 0 | 1 | 0 | 0 | 0 | 3,537.4 |
|  | 3,537.39 | 0.873 | 0.9 | 1 | 0 | 0 | 1 | 0 | 0 | 0 | 3,537.4 |
|  | 3,537.39 | 0.873 | 1 | 1 | 0 | 0 | 1 | 0 | 0 | 0 | 3,537.4 |
| SCPE-W | 3,537.39 | 0.873 | 0 | 1 | 0 | 0 | 1 | 0 | 0 | 0 | 3,537.4 |
|  | 3,537.39 | 0.873 | 0.1 | 1 | 0 | 0 | 1 | 0 | 0 | 0 | 3,537.4 |
|  | 3,537.39 | 0.873 | 0.2 | 1 | 0 | 0 | 1 | 0 | 0 | 0 | 3,537.4 |
|  | 3,537.39 | 0.873 | 0.3 | 1 | 0 | 0 | 1 | 0 | 0 | 0 | 3,537.4 |
|  | 3,537.39 | 0.873 | 0.4 | 1 | 0 | 0 | 1 | 0 | 0 | 0 | 3,537.4 |
|  | 3,537.39 | 0.873 | 0.5 | 1 | 0 | 0 | 1 | 0 | 0 | 0 | 3,537.4 |
|  | 3,537.39 | 0.873 | 0.6 | 1 | 0 | 0 | 1 | 0 | 0 | 0 | 3,537.4 |
|  | 3,537.39 | 0.873 | 0.7 | 1 | 0 | 0 | 1 | 0 | 0 | 0 | 3,537.4 |

Supporting Table S1. (continued)

| **Scenario** | **Yield (kg/ha)** | **q** | **p_o_** | **p_w_** | **p_s_** | **p_c_** | **^n^ANY** | **^n^PY** | **^n^OP** | **^n^SF** | **E(Y) (kg/ha)** |
| --- | --- | --- | --- | --- | --- | --- | --- | --- | --- | --- | --- |
|  | 3,537.39 | 0.873 | 0.8 | 1 | 0 | 0 | 1 | 0 | 0 | 0 | 3,537.4 |
|  | 3,537.39 | 0.873 | 0.9 | 1 | 0 | 0 | 1 | 0 | 0 | 0 | 3,537.4 |
|  | 3,537.39 | 0.873 | 1 | 1 | 0 | 0 | 1 | 0 | 0 | 0 | 3,537.4 |
| SCTM-W | 3,537.39 | 0.873 | 0 | 1 | 0 | 0 | 1 | 0 | 0 | 0 | 3,537.4 |
|  | 3,537.39 | 0.873 | 0.1 | 1 | 0 | 0 | 1 | 0 | 0 | 0 | 3,537.4 |
|  | 3,537.39 | 0.873 | 0.2 | 1 | 0 | 0 | 1 | 0 | 0 | 0 | 3,537.4 |
|  | 3,537.39 | 0.873 | 0.3 | 1 | 0 | 0 | 1 | 0 | 0 | 0 | 3,537.4 |
|  | 3,537.39 | 0.873 | 0.4 | 1 | 0 | 0 | 1 | 0 | 0 | 0 | 3,537.4 |
|  | 3,537.39 | 0.873 | 0.5 | 1 | 0 | 0 | 1 | 0 | 0 | 0 | 3,537.4 |
|  | 3,537.39 | 0.873 | 0.6 | 1 | 0 | 0 | 1 | 0 | 0 | 0 | 3,537.4 |
|  | 3,537.39 | 0.873 | 0.7 | 1 | 0 | 0 | 1 | 0 | 0 | 0 | 3,537.4 |
|  | 3,537.39 | 0.873 | 0.8 | 1 | 0 | 0 | 1 | 0 | 0 | 0 | 3,537.4 |
|  | 3,537.39 | 0.873 | 0.9 | 1 | 0 | 0 | 1 | 0 | 0 | 0 | 3,537.4 |
|  | 3,537.39 | 0.873 | 1 | 1 | 0 | 0 | 1 | 0 | 0 | 0 | 3,537.4 |
| RCXN-W | 3,537.39 | 1 | 0 | 1 | 0 | 0 | 0 | 0 | 0 | 0 | 3,537.4 |
|  | 3,537.39 | 1 | 0.1 | 1 | 0 | 0 | 0 | 0 | 0 | 0 | 3,537.4 |
|  | 3,537.39 | 1 | 0.2 | 1 | 0 | 0 | 0 | 0 | 0 | 0 | 3,537.4 |
|  | 3,537.39 | 1 | 0.3 | 1 | 0 | 0 | 0 | 0 | 0 | 0 | 3,537.4 |
|  | 3,537.39 | 1 | 0.4 | 1 | 0 | 0 | 0 | 0 | 0 | 0 | 3,537.4 |
|  | 3,537.39 | 1 | 0.5 | 1 | 0 | 0 | 0 | 0 | 0 | 0 | 3,537.4 |
|  | 3,537.39 | 1 | 0.6 | 1 | 0 | 0 | 0 | 0 | 0 | 0 | 3,537.4 |
|  | 3,537.39 | 1 | 0.7 | 1 | 0 | 0 | 0 | 0 | 0 | 0 | 3,537.4 |
|  | 3,537.39 | 1 | 0.8 | 1 | 0 | 0 | 0 | 0 | 0 | 0 | 3,537.4 |
|  | 3,537.39 | 1 | 0.9 | 1 | 0 | 0 | 0 | 0 | 0 | 0 | 3,537.4 |
|  | 3,537.39 | 1 | 1 | 1 | 0 | 0 | 0 | 0 | 0 | 0 | 3,537.4 |
| RCXM-W | 3,537.39 | 1 | 0 | 1 | 0 | 0 | 0 | 0 | 0 | 0 | 3,537.4 |
|  | 3,537.39 | 1 | 0.1 | 1 | 0 | 0 | 0 | 0 | 0 | 0 | 3,537.4 |
|  | 3,537.39 | 1 | 0.2 | 1 | 0 | 0 | 0 | 0 | 0 | 0 | 3,537.4 |

Supporting Table S1. (continued)

| **Scenario** | **Yield (kg/ha)** | **q** | **p_o_** | **p_w_** | **p_s_** | **p_c_** | **^n^ANY** | **^n^PY** | **^n^OP** | **^n^SF** | **E(Y) (kg/ha)** |
| --- | --- | --- | --- | --- | --- | --- | --- | --- | --- | --- | --- |
|  | 3,537.39 | 1 | 0.3 | 1 | 0 | 0 | 0 | 0 | 0 | 0 | 3,537.4 |
|  | 3,537.39 | 1 | 0.4 | 1 | 0 | 0 | 0 | 0 | 0 | 0 | 3,537.4 |
|  | 3,537.39 | 1 | 0.5 | 1 | 0 | 0 | 0 | 0 | 0 | 0 | 3,537.4 |
|  | 3,537.39 | 1 | 0.6 | 1 | 0 | 0 | 0 | 0 | 0 | 0 | 3,537.4 |
|  | 3,537.39 | 1 | 0.7 | 1 | 0 | 0 | 0 | 0 | 0 | 0 | 3,537.4 |
|  | 3,537.39 | 1 | 0.8 | 1 | 0 | 0 | 0 | 0 | 0 | 0 | 3,537.4 |
|  | 3,537.39 | 1 | 0.9 | 1 | 0 | 0 | 0 | 0 | 0 | 0 | 3,537.4 |
|  | 3,537.39 | 1 | 1 | 1 | 0 | 0 | 0 | 0 | 0 | 0 | 3,537.4 |
| SHXN-I | 3,537.39 | 0.873 | 0.4348 | 1 | 0 | 0 | 0 | 0 | 0 | 0 | 3,342.1 |
|  | 3,537.39 | 0.873 | 0.4348 | 0.9 | 0.1 | 0 | 0 | 0 | 0 | 0 | 3,342.1 |
|  | 3,537.39 | 0.873 | 0.4348 | 0.8 | 0.2 | 0 | 0 | 0 | 0 | 0 | 3,342.1 |
|  | 3,537.39 | 0.873 | 0.4348 | 0.7 | 0.3 | 0 | 0 | 0 | 0 | 0 | 3,342.1 |
|  | 3,537.39 | 0.873 | 0.4348 | 0.6 | 0.4 | 0 | 0 | 0 | 0 | 0 | 3,342.1 |
|  | 3,537.39 | 0.873 | 0.4348 | 0.5 | 0.5 | 0 | 0 | 0 | 0 | 0 | 3,342.1 |
|  | 3,537.39 | 0.873 | 0.4348 | 0.4 | 0.6 | 0 | 0 | 0 | 0 | 0 | 3,342.1 |
|  | 3,537.39 | 0.873 | 0.4348 | 0.3 | 0.7 | 0 | 0 | 0 | 0 | 0 | 3,342.1 |
|  | 3,537.39 | 0.873 | 0.4348 | 0.2 | 0.8 | 0 | 0 | 0 | 0 | 0 | 3,342.1 |
|  | 3,537.39 | 0.873 | 0.4348 | 0.1 | 0.9 | 0 | 0 | 0 | 0 | 0 | 3,342.1 |
|  | 3,537.39 | 0.873 | 0.4348 | 0 | 1 | 0 | 0 | 0 | 0 | 0 | 3,342.1 |
| SHPN-I | 3,537.39 | 0.873 | 0.4348 | 1 | 0 | 0 | 0 | 1 | 0 | 0 | 3,537.4 |
|  | 3,537.39 | 0.873 | 0.4348 | 0.9 | 0.1 | 0 | 0 | 1 | 0 | 0 | 3,517.9 |
|  | 3,537.39 | 0.873 | 0.4348 | 0.8 | 0.2 | 0 | 0 | 1 | 0 | 0 | 3,498.3 |
|  | 3,537.39 | 0.873 | 0.4348 | 0.7 | 0.3 | 0 | 0 | 1 | 0 | 0 | 3,478.8 |
|  | 3,537.39 | 0.873 | 0.4348 | 0.6 | 0.4 | 0 | 0 | 1 | 0 | 0 | 3,459.3 |
|  | 3,537.39 | 0.873 | 0.4348 | 0.5 | 0.5 | 0 | 0 | 1 | 0 | 0 | 3,439.7 |
|  | 3,537.39 | 0.873 | 0.4348 | 0.4 | 0.6 | 0 | 0 | 1 | 0 | 0 | 3,420.2 |
|  | 3,537.39 | 0.873 | 0.4348 | 0.3 | 0.7 | 0 | 0 | 1 | 0 | 0 | 3,400.7 |
|  | 3,537.39 | 0.873 | 0.4348 | 0.2 | 0.8 | 0 | 0 | 1 | 0 | 0 | 3,381.1 |

Supporting Table S1. (continued)

| **Scenario** | **Yield (kg/ha)** | **q** | **p_o_** | **p_w_** | **p_s_** | **p_c_** | **^n^ANY** | **^n^PY** | **^n^OP** | **^n^SF** | **E(Y) (kg/ha)** |
| --- | --- | --- | --- | --- | --- | --- | --- | --- | --- | --- | --- |
|  | 3,537.39 | 0.873 | 0.4348 | 0.1 | 0.9 | 0 | 0 | 1 | 0 | 0 | 3,361.6 |
|  | 3,537.39 | 0.873 | 0.4348 | 0 | 1 | 0 | 0 | 1 | 0 | 0 | 3,342.1 |
| SHPN-I | 3,537.39 | 0.873 | 0.4348 | 1 | 0 | 0 | 0 | 0 | 1 | 0 | 3,537.4 |
| (1B only) | 3,537.39 | 0.873 | 0.4348 | 0.9 | 0.1 | 0 | 0 | 0 | 1 | 0 | 3,537.4 |
|  | 3,537.39 | 0.873 | 0.4348 | 0.8 | 0.2 | 0 | 0 | 0 | 1 | 0 | 3,537.4 |
|  | 3,537.39 | 0.873 | 0.4348 | 0.7 | 0.3 | 0 | 0 | 0 | 1 | 0 | 3,537.4 |
|  | 3,537.39 | 0.873 | 0.4348 | 0.6 | 0.4 | 0 | 0 | 0 | 1 | 0 | 3,537.4 |
|  | 3,537.39 | 0.873 | 0.4348 | 0.5 | 0.5 | 0 | 0 | 0 | 1 | 0 | 3,537.4 |
|  | 3,537.39 | 0.873 | 0.4348 | 0.4 | 0.6 | 0 | 0 | 0 | 1 | 0 | 3,537.4 |
|  | 3,537.39 | 0.873 | 0.4348 | 0.3 | 0.7 | 0 | 0 | 0 | 1 | 0 | 3,537.4 |
|  | 3,537.39 | 0.873 | 0.4348 | 0.2 | 0.8 | 0 | 0 | 0 | 1 | 0 | 3,537.4 |
|  | 3,537.39 | 0.873 | 0.4348 | 0.1 | 0.9 | 0 | 0 | 0 | 1 | 0 | 3,537.4 |
|  | 3,537.39 | 0.873 | 0.4348 | 0 | 1 | 0 | 0 | 0 | 1 | 0 | 3,537.4 |
| SHPN-I | 3,537.39 | 0.873 | 0.4348 | 1 | 0 | 0 | 0 | 0 | 0 | 1 | 3,537.4 |
| (4C only) | 3,537.39 | 0.873 | 0.4348 | 0.9 | 0.1 | 0 | 0 | 0 | 0 | 1 | 3,537.4 |
|  | 3,537.39 | 0.873 | 0.4348 | 0.8 | 0.2 | 0 | 0 | 0 | 0 | 1 | 3,537.4 |
|  | 3,537.39 | 0.873 | 0.4348 | 0.7 | 0.3 | 0 | 0 | 0 | 0 | 1 | 3,537.4 |
|  | 3,537.39 | 0.873 | 0.4348 | 0.6 | 0.4 | 0 | 0 | 0 | 0 | 1 | 3,537.4 |
|  | 3,537.39 | 0.873 | 0.4348 | 0.5 | 0.5 | 0 | 0 | 0 | 0 | 1 | 3,537.4 |
|  | 3,537.39 | 0.873 | 0.4348 | 0.4 | 0.6 | 0 | 0 | 0 | 0 | 1 | 3,537.4 |
|  | 3,537.39 | 0.873 | 0.4348 | 0.3 | 0.7 | 0 | 0 | 0 | 0 | 1 | 3,537.4 |
|  | 3,537.39 | 0.873 | 0.4348 | 0.2 | 0.8 | 0 | 0 | 0 | 0 | 1 | 3,537.4 |
|  | 3,537.39 | 0.873 | 0.4348 | 0.1 | 0.9 | 0 | 0 | 0 | 0 | 1 | 3,537.4 |
|  | 3,537.39 | 0.873 | 0.4348 | 0 | 1 | 0 | 0 | 0 | 0 | 1 | 3,537.4 |
| SHPE-I | 3,537.39 | 0.873 | 0.4348 | 1 | 0 | 0 | 0 | 1 | 1 | 0 | 3,537.4 |
| (3A/1B) | 3,537.39 | 0.873 | 0.4348 | 0.9 | 0.1 | 0 | 0 | 1 | 1 | 0 | 3,537.4 |
|  | 3,537.39 | 0.873 | 0.4348 | 0.8 | 0.2 | 0 | 0 | 1 | 1 | 0 | 3,537.4 |
|  | 3,537.39 | 0.873 | 0.4348 | 0.7 | 0.3 | 0 | 0 | 1 | 1 | 0 | 3,537.4 |

Supporting Table S1. (continued)

| **Scenario** | **Yield (kg/ha)** | **q** | **p_o_** | **p_w_** | **p_s_** | **p_c_** | **^n^ANY** | **^n^PY** | **^n^OP** | **^n^SF** | **E(Y) (kg/ha)** |
| --- | --- | --- | --- | --- | --- | --- | --- | --- | --- | --- | --- |
|  | 3,537.39 | 0.873 | 0.4348 | 0.6 | 0.4 | 0 | 0 | 1 | 1 | 0 | 3,537.4 |
|  | 3,537.39 | 0.873 | 0.4348 | 0.5 | 0.5 | 0 | 0 | 1 | 1 | 0 | 3,537.4 |
|  | 3,537.39 | 0.873 | 0.4348 | 0.4 | 0.6 | 0 | 0 | 1 | 1 | 0 | 3,537.4 |
|  | 3,537.39 | 0.873 | 0.4348 | 0.3 | 0.7 | 0 | 0 | 1 | 1 | 0 | 3,537.4 |
|  | 3,537.39 | 0.873 | 0.4348 | 0.2 | 0.8 | 0 | 0 | 1 | 1 | 0 | 3,537.4 |
|  | 3,537.39 | 0.873 | 0.4348 | 0.1 | 0.9 | 0 | 0 | 1 | 1 | 0 | 3,537.4 |
|  | 3,537.39 | 0.873 | 0.4348 | 0 | 1 | 0 | 0 | 1 | 1 | 0 | 3,537.4 |
| SHPE-I | 3,537.39 | 0.873 | 0.4348 | 1 | 0 | 0 | 0 | 0 | 0 | 1 | 3,537.4 |
| (4C only) | 3,537.39 | 0.873 | 0.4348 | 0.9 | 0.1 | 0 | 0 | 0 | 0 | 1 | 3,537.4 |
|  | 3,537.39 | 0.873 | 0.4348 | 0.8 | 0.2 | 0 | 0 | 0 | 0 | 1 | 3,537.4 |
|  | 3,537.39 | 0.873 | 0.4348 | 0.7 | 0.3 | 0 | 0 | 0 | 0 | 1 | 3,537.4 |
|  | 3,537.39 | 0.873 | 0.4348 | 0.6 | 0.4 | 0 | 0 | 0 | 0 | 1 | 3,537.4 |
|  | 3,537.39 | 0.873 | 0.4348 | 0.5 | 0.5 | 0 | 0 | 0 | 0 | 1 | 3,537.4 |
|  | 3,537.39 | 0.873 | 0.4348 | 0.4 | 0.6 | 0 | 0 | 0 | 0 | 1 | 3,537.4 |
|  | 3,537.39 | 0.873 | 0.4348 | 0.3 | 0.7 | 0 | 0 | 0 | 0 | 1 | 3,537.4 |
|  | 3,537.39 | 0.873 | 0.4348 | 0.2 | 0.8 | 0 | 0 | 0 | 0 | 1 | 3,537.4 |
|  | 3,537.39 | 0.873 | 0.4348 | 0.1 | 0.9 | 0 | 0 | 0 | 0 | 1 | 3,537.4 |
|  | 3,537.39 | 0.873 | 0.4348 | 0 | 1 | 0 | 0 | 0 | 0 | 1 | 3,537.4 |
| SHPN-I | 3,537.39 | 0.873 | 0.4348 | 1 | 0 | 0 | 0 | 0 | 0 | 1 | 3,537.4 |
| (4C only) | 3,537.39 | 0.873 | 0.4348 | 0.9 | 0 | 0.1 | 0 | 0 | 0 | 1 | 3,537.4 |
|  | 3,537.39 | 0.873 | 0.4348 | 0.8 | 0 | 0.2 | 0 | 0 | 0 | 1 | 3,537.4 |
|  | 3,537.39 | 0.873 | 0.4348 | 0.7 | 0 | 0.3 | 0 | 0 | 0 | 1 | 3,537.4 |
|  | 3,537.39 | 0.873 | 0.4348 | 0.6 | 0 | 0.4 | 0 | 0 | 0 | 1 | 3,537.4 |
|  | 3,537.39 | 0.873 | 0.4348 | 0.5 | 0 | 0.5 | 0 | 0 | 0 | 1 | 3,537.4 |
|  | 3,537.39 | 0.873 | 0.4348 | 0.4 | 0 | 0.6 | 0 | 0 | 0 | 1 | 3,537.4 |
|  | 3,537.39 | 0.873 | 0.4348 | 0.3 | 0 | 0.7 | 0 | 0 | 0 | 1 | 3,537.4 |
|  | 3,537.39 | 0.873 | 0.4348 | 0.2 | 0 | 0.8 | 0 | 0 | 0 | 1 | 3,537.4 |
|  | 3,537.39 | 0.873 | 0.4348 | 0.1 | 0 | 0.9 | 0 | 0 | 0 | 1 | 3,537.4 |

Supporting Table S1. (continued)

| **Scenario** | **Yield (kg/ha)** | **q** | **p_o_** | **p_w_** | **p_s_** | **p_c_** | **^n^ANY** | **^n^PY** | **^n^OP** | **^n^SF** | **E(Y) (kg/ha)** |
| --- | --- | --- | --- | --- | --- | --- | --- | --- | --- | --- | --- |
|  | 3,537.39 | 0.873 | 0.4348 | 0 | 0 | 1 | 0 | 0 | 0 | 1 | 3,537.4 |
| SHPE-I | 3,537.39 | 0.873 | 0.4348 | 1 | 0 | 0 | 0 | 0 | 1 | 1 | 3,537.4 |
| (1B/4C) | 3,537.39 | 0.873 | 0.4348 | 0.9 | 0 | 0.1 | 0 | 0 | 1 | 1 | 3,537.4 |
|  | 3,537.39 | 0.873 | 0.4348 | 0.8 | 0 | 0.2 | 0 | 0 | 1 | 1 | 3,537.4 |
|  | 3,537.39 | 0.873 | 0.4348 | 0.7 | 0 | 0.3 | 0 | 0 | 1 | 1 | 3,537.4 |
|  | 3,537.39 | 0.873 | 0.4348 | 0.6 | 0 | 0.4 | 0 | 0 | 1 | 1 | 3,537.4 |
|  | 3,537.39 | 0.873 | 0.4348 | 0.5 | 0 | 0.5 | 0 | 0 | 1 | 1 | 3,537.4 |
|  | 3,537.39 | 0.873 | 0.4348 | 0.4 | 0 | 0.6 | 0 | 0 | 1 | 1 | 3,537.4 |
|  | 3,537.39 | 0.873 | 0.4348 | 0.3 | 0 | 0.7 | 0 | 0 | 1 | 1 | 3,537.4 |
|  | 3,537.39 | 0.873 | 0.4348 | 0.2 | 0 | 0.8 | 0 | 0 | 1 | 1 | 3,537.4 |
|  | 3,537.39 | 0.873 | 0.4348 | 0.1 | 0 | 0.9 | 0 | 0 | 1 | 1 | 3,537.4 |
|  | 3,537.39 | 0.873 | 0.4348 | 0 | 0 | 1 | 0 | 0 | 1 | 1 | 3,537.4 |
| SHTM-I | 3,537.39 | 0.873 | 0.4348 | 1 | 0 | 0 | 0 | 1 | 1 | 0 | 3,537.4 |
| (3A/1B) | 3,537.39 | 0.873 | 0.4348 | 0.9 | 0.1 | 0 | 0 | 1 | 1 | 0 | 3,537.4 |
|  | 3,537.39 | 0.873 | 0.4348 | 0.8 | 0.2 | 0 | 0 | 1 | 1 | 0 | 3,537.4 |
|  | 3,537.39 | 0.873 | 0.4348 | 0.7 | 0.3 | 0 | 0 | 1 | 1 | 0 | 3,537.4 |
|  | 3,537.39 | 0.873 | 0.4348 | 0.6 | 0.4 | 0 | 0 | 1 | 1 | 0 | 3,537.4 |
|  | 3,537.39 | 0.873 | 0.4348 | 0.5 | 0.5 | 0 | 0 | 1 | 1 | 0 | 3,537.4 |
|  | 3,537.39 | 0.873 | 0.4348 | 0.4 | 0.6 | 0 | 0 | 1 | 1 | 0 | 3,537.4 |
|  | 3,537.39 | 0.873 | 0.4348 | 0.3 | 0.7 | 0 | 0 | 1 | 1 | 0 | 3,537.4 |
|  | 3,537.39 | 0.873 | 0.4348 | 0.2 | 0.8 | 0 | 0 | 1 | 1 | 0 | 3,537.4 |
|  | 3,537.39 | 0.873 | 0.4348 | 0.1 | 0.9 | 0 | 0 | 1 | 1 | 0 | 3,537.4 |
|  | 3,537.39 | 0.873 | 0.4348 | 0 | 1 | 0 | 0 | 1 | 1 | 0 | 3,537.4 |
| SHTM-I | 3,537.39 | 0.873 | 0.4348 | 1 | 0 | 0 | 0 | 0 | 1 | 0 | 3,537.4 |
| (1B only) | 3,537.39 | 0.873 | 0.4348 | 0.9 | 0.1 | 0 | 0 | 0 | 1 | 0 | 3,537.4 |
|  | 3,537.39 | 0.873 | 0.4348 | 0.8 | 0.2 | 0 | 0 | 0 | 1 | 0 | 3,537.4 |
|  | 3,537.39 | 0.873 | 0.4348 | 0.7 | 0.3 | 0 | 0 | 0 | 1 | 0 | 3,537.4 |
|  | 3,537.39 | 0.873 | 0.4348 | 0.6 | 0.4 | 0 | 0 | 0 | 1 | 0 | 3,537.4 |

Supporting Table S1. (continued)

| **Scenario** | **Yield (kg/ha)** | **q** | **p_o_** | **p_w_** | **p_s_** | **p_c_** | **^n^ANY** | **^n^PY** | **^n^OP** | **^n^SF** | **E(Y) (kg/ha)** |
| --- | --- | --- | --- | --- | --- | --- | --- | --- | --- | --- | --- |
|  | 3,537.39 | 0.873 | 0.4348 | 0.5 | 0.5 | 0 | 0 | 0 | 1 | 0 | 3,537.4 |
|  | 3,537.39 | 0.873 | 0.4348 | 0.4 | 0.6 | 0 | 0 | 0 | 1 | 0 | 3,537.4 |
|  | 3,537.39 | 0.873 | 0.4348 | 0.3 | 0.7 | 0 | 0 | 0 | 1 | 0 | 3,537.4 |
|  | 3,537.39 | 0.873 | 0.4348 | 0.2 | 0.8 | 0 | 0 | 0 | 1 | 0 | 3,537.4 |
|  | 3,537.39 | 0.873 | 0.4348 | 0.1 | 0.9 | 0 | 0 | 0 | 1 | 0 | 3,537.4 |
|  | 3,537.39 | 0.873 | 0.4348 | 0 | 1 | 0 | 0 | 0 | 1 | 0 | 3,537.4 |
| SHTM-I | 3,537.39 | 0.873 | 0.4348 | 1 | 0 | 0 | 0 | 0 | 0 | 1 | 3,537.4 |
| (4C only) | 3,537.39 | 0.873 | 0.4348 | 0.9 | 0.1 | 0 | 0 | 0 | 0 | 1 | 3,537.4 |
|  | 3,537.39 | 0.873 | 0.4348 | 0.8 | 0.2 | 0 | 0 | 0 | 0 | 1 | 3,537.4 |
|  | 3,537.39 | 0.873 | 0.4348 | 0.7 | 0.3 | 0 | 0 | 0 | 0 | 1 | 3,537.4 |
|  | 3,537.39 | 0.873 | 0.4348 | 0.6 | 0.4 | 0 | 0 | 0 | 0 | 1 | 3,537.4 |
|  | 3,537.39 | 0.873 | 0.4348 | 0.5 | 0.5 | 0 | 0 | 0 | 0 | 1 | 3,537.4 |
|  | 3,537.39 | 0.873 | 0.4348 | 0.4 | 0.6 | 0 | 0 | 0 | 0 | 1 | 3,537.4 |
|  | 3,537.39 | 0.873 | 0.4348 | 0.3 | 0.7 | 0 | 0 | 0 | 0 | 1 | 3,537.4 |
|  | 3,537.39 | 0.873 | 0.4348 | 0.2 | 0.8 | 0 | 0 | 0 | 0 | 1 | 3,537.4 |
|  | 3,537.39 | 0.873 | 0.4348 | 0.1 | 0.9 | 0 | 0 | 0 | 0 | 1 | 3,537.4 |
|  | 3,537.39 | 0.873 | 0.4348 | 0 | 1 | 0 | 0 | 0 | 0 | 1 | 3,537.4 |
| SHTM-I | 3,537.39 | 0.873 | 0.4348 | 1 | 0 | 0 | 0 | 0 | 1 | 1 | 3,537.4 |
| (1B/4C) | 3,537.39 | 0.873 | 0.4348 | 0.9 | 0 | 0.1 | 0 | 0 | 1 | 1 | 3,537.4 |
|  | 3,537.39 | 0.873 | 0.4348 | 0.8 | 0 | 0.2 | 0 | 0 | 1 | 1 | 3,537.4 |
|  | 3,537.39 | 0.873 | 0.4348 | 0.7 | 0 | 0.3 | 0 | 0 | 1 | 1 | 3,537.4 |
|  | 3,537.39 | 0.873 | 0.4348 | 0.6 | 0 | 0.4 | 0 | 0 | 1 | 1 | 3,537.4 |
|  | 3,537.39 | 0.873 | 0.4348 | 0.5 | 0 | 0.5 | 0 | 0 | 1 | 1 | 3,537.4 |
|  | 3,537.39 | 0.873 | 0.4348 | 0.4 | 0 | 0.6 | 0 | 0 | 1 | 1 | 3,537.4 |
|  | 3,537.39 | 0.873 | 0.4348 | 0.3 | 0 | 0.7 | 0 | 0 | 1 | 1 | 3,537.4 |
|  | 3,537.39 | 0.873 | 0.4348 | 0.2 | 0 | 0.8 | 0 | 0 | 1 | 1 | 3,537.4 |
|  | 3,537.39 | 0.873 | 0.4348 | 0.1 | 0 | 0.9 | 0 | 0 | 1 | 1 | 3,537.4 |
|  | 3,537.39 | 0.873 | 0.4348 | 0 | 0 | 1 | 0 | 0 | 1 | 1 | 3,537.4 |

Supporting Table S1. (continued)

| **Scenario** | **Yield (kg/ha)** | **q** | **p_o_** | **p_w_** | **p_s_** | **p_c_** | **^n^ANY** | **^n^PY** | **^n^OP** | **^n^SF** | **E(Y) (kg/ha)** |
| --- | --- | --- | --- | --- | --- | --- | --- | --- | --- | --- | --- |
| SHTM-I | 3,537.39 | 0.873 | 0.4348 | 1 | 0 | 0 | 0 | 0 | 0 | 1 | 3,537.4 |
| (4C only) | 3,537.39 | 0.873 | 0.4348 | 0.9 | 0 | 0.1 | 0 | 0 | 0 | 1 | 3,537.4 |
|  | 3,537.39 | 0.873 | 0.4348 | 0.8 | 0 | 0.2 | 0 | 0 | 0 | 1 | 3,537.4 |
|  | 3,537.39 | 0.873 | 0.4348 | 0.7 | 0 | 0.3 | 0 | 0 | 0 | 1 | 3,537.4 |
|  | 3,537.39 | 0.873 | 0.4348 | 0.6 | 0 | 0.4 | 0 | 0 | 0 | 1 | 3,537.4 |
|  | 3,537.39 | 0.873 | 0.4348 | 0.5 | 0 | 0.5 | 0 | 0 | 0 | 1 | 3,537.4 |
|  | 3,537.39 | 0.873 | 0.4348 | 0.4 | 0 | 0.6 | 0 | 0 | 0 | 1 | 3,537.4 |
|  | 3,537.39 | 0.873 | 0.4348 | 0.3 | 0 | 0.7 | 0 | 0 | 0 | 1 | 3,537.4 |
|  | 3,537.39 | 0.873 | 0.4348 | 0.2 | 0 | 0.8 | 0 | 0 | 0 | 1 | 3,537.4 |
|  | 3,537.39 | 0.873 | 0.4348 | 0.1 | 0 | 0.9 | 0 | 0 | 0 | 1 | 3,537.4 |
|  | 3,537.39 | 0.873 | 0.4348 | 0 | 0 | 1 | 0 | 0 | 0 | 1 | 3,537.4 |
| SCXN-I | 3,537.39 | 0.873 | 0.4348 | 1 | 0 | 0 | 0 | 0 | 0 | 0 | 3,342.1 |
|  | 3,537.39 | 0.873 | 0.4348 | 0.9 | 0.1 | 0 | 0 | 0 | 0 | 0 | 3,342.1 |
|  | 3,537.39 | 0.873 | 0.4348 | 0.8 | 0.2 | 0 | 0 | 0 | 0 | 0 | 3,342.1 |
|  | 3,537.39 | 0.873 | 0.4348 | 0.7 | 0.3 | 0 | 0 | 0 | 0 | 0 | 3,342.1 |
|  | 3,537.39 | 0.873 | 0.4348 | 0.6 | 0.4 | 0 | 0 | 0 | 0 | 0 | 3,342.1 |
|  | 3,537.39 | 0.873 | 0.4348 | 0.5 | 0.5 | 0 | 0 | 0 | 0 | 0 | 3,342.1 |
|  | 3,537.39 | 0.873 | 0.4348 | 0.4 | 0.6 | 0 | 0 | 0 | 0 | 0 | 3,342.1 |
|  | 3,537.39 | 0.873 | 0.4348 | 0.3 | 0.7 | 0 | 0 | 0 | 0 | 0 | 3,342.1 |
|  | 3,537.39 | 0.873 | 0.4348 | 0.2 | 0.8 | 0 | 0 | 0 | 0 | 0 | 3,342.1 |
|  | 3,537.39 | 0.873 | 0.4348 | 0.1 | 0.9 | 0 | 0 | 0 | 0 | 0 | 3,342.1 |
|  | 3,537.39 | 0.873 | 0.4348 | 0 | 1 | 0 | 0 | 0 | 0 | 0 | 3,342.1 |
| SCPN-I | 3,537.39 | 0.873 | 0.4348 | 1 | 0 | 0 | 0 | 1 | 0 | 0 | 3,537.4 |
|  | 3,537.39 | 0.873 | 0.4348 | 0.9 | 0.1 | 0 | 0 | 1 | 0 | 0 | 3,517.9 |
|  | 3,537.39 | 0.873 | 0.4348 | 0.8 | 0.2 | 0 | 0 | 1 | 0 | 0 | 3,498.3 |
|  | 3,537.39 | 0.873 | 0.4348 | 0.7 | 0.3 | 0 | 0 | 1 | 0 | 0 | 3,478.8 |
|  | 3,537.39 | 0.873 | 0.4348 | 0.6 | 0.4 | 0 | 0 | 1 | 0 | 0 | 3,459.3 |
|  | 3,537.39 | 0.873 | 0.4348 | 0.5 | 0.5 | 0 | 0 | 1 | 0 | 0 | 3,439.7 |

Supporting Table S1. (continued)

| **Scenario** | **Yield (kg/ha)** | **q** | **p_o_** | **p_w_** | **p_s_** | **p_c_** | **^n^ANY** | **^n^PY** | **^n^OP** | **^n^SF** | **E(Y) (kg/ha)** |
| --- | --- | --- | --- | --- | --- | --- | --- | --- | --- | --- | --- |
|  | 3,537.39 | 0.873 | 0.4348 | 0.4 | 0.6 | 0 | 0 | 1 | 0 | 0 | 3,420.2 |
|  | 3,537.39 | 0.873 | 0.4348 | 0.3 | 0.7 | 0 | 0 | 1 | 0 | 0 | 3,400.7 |
|  | 3,537.39 | 0.873 | 0.4348 | 0.2 | 0.8 | 0 | 0 | 1 | 0 | 0 | 3,381.1 |
|  | 3,537.39 | 0.873 | 0.4348 | 0.1 | 0.9 | 0 | 0 | 1 | 0 | 0 | 3,361.6 |
|  | 3,537.39 | 0.873 | 0.4348 | 0 | 1 | 0 | 0 | 1 | 0 | 0 | 3,342.1 |
| SCPN-I | 3,537.39 | 0.873 | 0.4348 | 1 | 0 | 0 | 0 | 0 | 1 | 0 | 3,537.4 |
| (1B only) | 3,537.39 | 0.873 | 0.4348 | 0.9 | 0.1 | 0 | 0 | 0 | 1 | 0 | 3,537.4 |
|  | 3,537.39 | 0.873 | 0.4348 | 0.8 | 0.2 | 0 | 0 | 0 | 1 | 0 | 3,537.4 |
|  | 3,537.39 | 0.873 | 0.4348 | 0.7 | 0.3 | 0 | 0 | 0 | 1 | 0 | 3,537.4 |
|  | 3,537.39 | 0.873 | 0.4348 | 0.6 | 0.4 | 0 | 0 | 0 | 1 | 0 | 3,537.4 |
|  | 3,537.39 | 0.873 | 0.4348 | 0.5 | 0.5 | 0 | 0 | 0 | 1 | 0 | 3,537.4 |
|  | 3,537.39 | 0.873 | 0.4348 | 0.4 | 0.6 | 0 | 0 | 0 | 1 | 0 | 3,537.4 |
|  | 3,537.39 | 0.873 | 0.4348 | 0.3 | 0.7 | 0 | 0 | 0 | 1 | 0 | 3,537.4 |
|  | 3,537.39 | 0.873 | 0.4348 | 0.2 | 0.8 | 0 | 0 | 0 | 1 | 0 | 3,537.4 |
|  | 3,537.39 | 0.873 | 0.4348 | 0.1 | 0.9 | 0 | 0 | 0 | 1 | 0 | 3,537.4 |
|  | 3,537.39 | 0.873 | 0.4348 | 0 | 1 | 0 | 0 | 0 | 1 | 0 | 3,537.4 |
| SCPN-I | 3,537.39 | 0.873 | 0.4348 | 1 | 0 | 0 | 0 | 0 | 0 | 1 | 3,537.4 |
| (4C only) | 3,537.39 | 0.873 | 0.4348 | 0.9 | 0.1 | 0 | 0 | 0 | 0 | 1 | 3,537.4 |
|  | 3,537.39 | 0.873 | 0.4348 | 0.8 | 0.2 | 0 | 0 | 0 | 0 | 1 | 3,537.4 |
|  | 3,537.39 | 0.873 | 0.4348 | 0.7 | 0.3 | 0 | 0 | 0 | 0 | 1 | 3,537.4 |
|  | 3,537.39 | 0.873 | 0.4348 | 0.6 | 0.4 | 0 | 0 | 0 | 0 | 1 | 3,537.4 |
|  | 3,537.39 | 0.873 | 0.4348 | 0.5 | 0.5 | 0 | 0 | 0 | 0 | 1 | 3,537.4 |
|  | 3,537.39 | 0.873 | 0.4348 | 0.4 | 0.6 | 0 | 0 | 0 | 0 | 1 | 3,537.4 |
|  | 3,537.39 | 0.873 | 0.4348 | 0.3 | 0.7 | 0 | 0 | 0 | 0 | 1 | 3,537.4 |
|  | 3,537.39 | 0.873 | 0.4348 | 0.2 | 0.8 | 0 | 0 | 0 | 0 | 1 | 3,537.4 |
|  | 3,537.39 | 0.873 | 0.4348 | 0.1 | 0.9 | 0 | 0 | 0 | 0 | 1 | 3,537.4 |
|  | 3,537.39 | 0.873 | 0.4348 | 0 | 1 | 0 | 0 | 0 | 0 | 1 | 3,537.4 |

Supporting Table S1. (continued)

| **Scenario** | **Yield (kg/ha)** | **q** | **p_o_** | **p_w_** | **p_s_** | **p_c_** | **^n^ANY** | **^n^PY** | **^n^OP** | **^n^SF** | **E(Y) (kg/ha)** |
| --- | --- | --- | --- | --- | --- | --- | --- | --- | --- | --- | --- |
| SCPE-I | 3,537.39 | 0.873 | 0.4348 | 1 | 0 | 0 | 0 | 1 | 1 | 0 | 3,537.4 |
| (3A/1B) | 3,537.39 | 0.873 | 0.4348 | 0.9 | 0.1 | 0 | 0 | 1 | 1 | 0 | 3,537.4 |
|  | 3,537.39 | 0.873 | 0.4348 | 0.8 | 0.2 | 0 | 0 | 1 | 1 | 0 | 3,537.4 |
|  | 3,537.39 | 0.873 | 0.4348 | 0.7 | 0.3 | 0 | 0 | 1 | 1 | 0 | 3,537.4 |
|  | 3,537.39 | 0.873 | 0.4348 | 0.6 | 0.4 | 0 | 0 | 1 | 1 | 0 | 3,537.4 |
|  | 3,537.39 | 0.873 | 0.4348 | 0.5 | 0.5 | 0 | 0 | 1 | 1 | 0 | 3,537.4 |
|  | 3,537.39 | 0.873 | 0.4348 | 0.4 | 0.6 | 0 | 0 | 1 | 1 | 0 | 3,537.4 |
|  | 3,537.39 | 0.873 | 0.4348 | 0.3 | 0.7 | 0 | 0 | 1 | 1 | 0 | 3,537.4 |
|  | 3,537.39 | 0.873 | 0.4348 | 0.2 | 0.8 | 0 | 0 | 1 | 1 | 0 | 3,537.4 |
|  | 3,537.39 | 0.873 | 0.4348 | 0.1 | 0.9 | 0 | 0 | 1 | 1 | 0 | 3,537.4 |
|  | 3,537.39 | 0.873 | 0.4348 | 0 | 1 | 0 | 0 | 1 | 1 | 0 | 3,537.4 |
| SCPE-I | 3,537.39 | 0.873 | 0.4348 | 1 | 0 | 0 | 0 | 0 | 0 | 1 | 3,537.4 |
| (4C only) | 3,537.39 | 0.873 | 0.4348 | 0.9 | 0.1 | 0 | 0 | 0 | 0 | 1 | 3,537.4 |
|  | 3,537.39 | 0.873 | 0.4348 | 0.8 | 0.2 | 0 | 0 | 0 | 0 | 1 | 3,537.4 |
|  | 3,537.39 | 0.873 | 0.4348 | 0.7 | 0.3 | 0 | 0 | 0 | 0 | 1 | 3,537.4 |
|  | 3,537.39 | 0.873 | 0.4348 | 0.6 | 0.4 | 0 | 0 | 0 | 0 | 1 | 3,537.4 |
|  | 3,537.39 | 0.873 | 0.4348 | 0.5 | 0.5 | 0 | 0 | 0 | 0 | 1 | 3,537.4 |
|  | 3,537.39 | 0.873 | 0.4348 | 0.4 | 0.6 | 0 | 0 | 0 | 0 | 1 | 3,537.4 |
|  | 3,537.39 | 0.873 | 0.4348 | 0.3 | 0.7 | 0 | 0 | 0 | 0 | 1 | 3,537.4 |
|  | 3,537.39 | 0.873 | 0.4348 | 0.2 | 0.8 | 0 | 0 | 0 | 0 | 1 | 3,537.4 |
|  | 3,537.39 | 0.873 | 0.4348 | 0.1 | 0.9 | 0 | 0 | 0 | 0 | 1 | 3,537.4 |
|  | 3,537.39 | 0.873 | 0.4348 | 0 | 1 | 0 | 0 | 0 | 0 | 1 | 3,537.4 |
| SCPN-I | 3,537.39 | 0.873 | 0.4348 | 1 | 0 | 0 | 0 | 0 | 0 | 1 | 3,537.4 |
| (4C only) | 3,537.39 | 0.873 | 0.4348 | 0.9 | 0 | 0.1 | 0 | 0 | 0 | 1 | 3,537.4 |
|  | 3,537.39 | 0.873 | 0.4348 | 0.8 | 0 | 0.2 | 0 | 0 | 0 | 1 | 3,537.4 |
|  | 3,537.39 | 0.873 | 0.4348 | 0.7 | 0 | 0.3 | 0 | 0 | 0 | 1 | 3,537.4 |
|  | 3,537.39 | 0.873 | 0.4348 | 0.6 | 0 | 0.4 | 0 | 0 | 0 | 1 | 3,537.4 |
|  | 3,537.39 | 0.873 | 0.4348 | 0.5 | 0 | 0.5 | 0 | 0 | 0 | 1 | 3,537.4 |

Supporting Table S1. (continued)

| **Scenario** | **Yield (kg/ha)** | **q** | **p_o_** | **p_w_** | **p_s_** | **p_c_** | **^n^ANY** | **^n^PY** | **^n^OP** | **^n^SF** | **E(Y) (kg/ha)** |
| --- | --- | --- | --- | --- | --- | --- | --- | --- | --- | --- | --- |
|  | 3,537.39 | 0.873 | 0.4348 | 0.4 | 0 | 0.6 | 0 | 0 | 0 | 1 | 3,537.4 |
|  | 3,537.39 | 0.873 | 0.4348 | 0.3 | 0 | 0.7 | 0 | 0 | 0 | 1 | 3,537.4 |
|  | 3,537.39 | 0.873 | 0.4348 | 0.2 | 0 | 0.8 | 0 | 0 | 0 | 1 | 3,537.4 |
|  | 3,537.39 | 0.873 | 0.4348 | 0.1 | 0 | 0.9 | 0 | 0 | 0 | 1 | 3,537.4 |
|  | 3,537.39 | 0.873 | 0.4348 | 0 | 0 | 1 | 0 | 0 | 0 | 1 | 3,537.4 |
| SCPE-I | 3,537.39 | 0.873 | 0.4348 | 1 | 0 | 0 | 0 | 0 | 1 | 1 | 3,537.4 |
| (1B/4C) | 3,537.39 | 0.873 | 0.4348 | 0.9 | 0 | 0.1 | 0 | 0 | 1 | 1 | 3,537.4 |
|  | 3,537.39 | 0.873 | 0.4348 | 0.8 | 0 | 0.2 | 0 | 0 | 1 | 1 | 3,537.4 |
|  | 3,537.39 | 0.873 | 0.4348 | 0.7 | 0 | 0.3 | 0 | 0 | 1 | 1 | 3,537.4 |
|  | 3,537.39 | 0.873 | 0.4348 | 0.6 | 0 | 0.4 | 0 | 0 | 1 | 1 | 3,537.4 |
|  | 3,537.39 | 0.873 | 0.4348 | 0.5 | 0 | 0.5 | 0 | 0 | 1 | 1 | 3,537.4 |
|  | 3,537.39 | 0.873 | 0.4348 | 0.4 | 0 | 0.6 | 0 | 0 | 1 | 1 | 3,537.4 |
|  | 3,537.39 | 0.873 | 0.4348 | 0.3 | 0 | 0.7 | 0 | 0 | 1 | 1 | 3,537.4 |
|  | 3,537.39 | 0.873 | 0.4348 | 0.2 | 0 | 0.8 | 0 | 0 | 1 | 1 | 3,537.4 |
|  | 3,537.39 | 0.873 | 0.4348 | 0.1 | 0 | 0.9 | 0 | 0 | 1 | 1 | 3,537.4 |
|  | 3,537.39 | 0.873 | 0.4348 | 0 | 0 | 1 | 0 | 0 | 1 | 1 | 3,537.4 |
| SCTM-I | 3,537.39 | 0.873 | 0.4348 | 1 | 0 | 0 | 0 | 1 | 1 | 0 | 3,537.4 |
| (3A/1B) | 3,537.39 | 0.873 | 0.4348 | 0.9 | 0.1 | 0 | 0 | 1 | 1 | 0 | 3,537.4 |
|  | 3,537.39 | 0.873 | 0.4348 | 0.8 | 0.2 | 0 | 0 | 1 | 1 | 0 | 3,537.4 |
|  | 3,537.39 | 0.873 | 0.4348 | 0.7 | 0.3 | 0 | 0 | 1 | 1 | 0 | 3,537.4 |
|  | 3,537.39 | 0.873 | 0.4348 | 0.6 | 0.4 | 0 | 0 | 1 | 1 | 0 | 3,537.4 |
|  | 3,537.39 | 0.873 | 0.4348 | 0.5 | 0.5 | 0 | 0 | 1 | 1 | 0 | 3,537.4 |
|  | 3,537.39 | 0.873 | 0.4348 | 0.4 | 0.6 | 0 | 0 | 1 | 1 | 0 | 3,537.4 |
|  | 3,537.39 | 0.873 | 0.4348 | 0.3 | 0.7 | 0 | 0 | 1 | 1 | 0 | 3,537.4 |
|  | 3,537.39 | 0.873 | 0.4348 | 0.2 | 0.8 | 0 | 0 | 1 | 1 | 0 | 3,537.4 |
|  | 3,537.39 | 0.873 | 0.4348 | 0.1 | 0.9 | 0 | 0 | 1 | 1 | 0 | 3,537.4 |
|  | 3,537.39 | 0.873 | 0.4348 | 0 | 1 | 0 | 0 | 1 | 1 | 0 | 3,537.4 |

Supporting Table S1. (continued)

| **Scenario** | **Yield (kg/ha)** | **q** | **p_o_** | **p_w_** | **p_s_** | **p_c_** | **^n^ANY** | **^n^PY** | **^n^OP** | **^n^SF** | **E(Y) (kg/ha)** |
| --- | --- | --- | --- | --- | --- | --- | --- | --- | --- | --- | --- |
| SCTM-I | 3,537.39 | 0.873 | 0.4348 | 1 | 0 | 0 | 0 | 0 | 1 | 0 | 3,537.4 |
| (1B only) | 3,537.39 | 0.873 | 0.4348 | 0.9 | 0.1 | 0 | 0 | 0 | 1 | 0 | 3,537.4 |
|  | 3,537.39 | 0.873 | 0.4348 | 0.8 | 0.2 | 0 | 0 | 0 | 1 | 0 | 3,537.4 |
|  | 3,537.39 | 0.873 | 0.4348 | 0.7 | 0.3 | 0 | 0 | 0 | 1 | 0 | 3,537.4 |
|  | 3,537.39 | 0.873 | 0.4348 | 0.6 | 0.4 | 0 | 0 | 0 | 1 | 0 | 3,537.4 |
|  | 3,537.39 | 0.873 | 0.4348 | 0.5 | 0.5 | 0 | 0 | 0 | 1 | 0 | 3,537.4 |
|  | 3,537.39 | 0.873 | 0.4348 | 0.4 | 0.6 | 0 | 0 | 0 | 1 | 0 | 3,537.4 |
|  | 3,537.39 | 0.873 | 0.4348 | 0.3 | 0.7 | 0 | 0 | 0 | 1 | 0 | 3,537.4 |
|  | 3,537.39 | 0.873 | 0.4348 | 0.2 | 0.8 | 0 | 0 | 0 | 1 | 0 | 3,537.4 |
|  | 3,537.39 | 0.873 | 0.4348 | 0.1 | 0.9 | 0 | 0 | 0 | 1 | 0 | 3,537.4 |
|  | 3,537.39 | 0.873 | 0.4348 | 0 | 1 | 0 | 0 | 0 | 1 | 0 | 3,537.4 |
| SCTM-I | 3,537.39 | 0.873 | 0.4348 | 1 | 0 | 0 | 0 | 0 | 0 | 1 | 3,537.4 |
| (4C only) | 3,537.39 | 0.873 | 0.4348 | 0.9 | 0.1 | 0 | 0 | 0 | 0 | 1 | 3,537.4 |
|  | 3,537.39 | 0.873 | 0.4348 | 0.8 | 0.2 | 0 | 0 | 0 | 0 | 1 | 3,537.4 |
|  | 3,537.39 | 0.873 | 0.4348 | 0.7 | 0.3 | 0 | 0 | 0 | 0 | 1 | 3,537.4 |
|  | 3,537.39 | 0.873 | 0.4348 | 0.6 | 0.4 | 0 | 0 | 0 | 0 | 1 | 3,537.4 |
|  | 3,537.39 | 0.873 | 0.4348 | 0.5 | 0.5 | 0 | 0 | 0 | 0 | 1 | 3,537.4 |
|  | 3,537.39 | 0.873 | 0.4348 | 0.4 | 0.6 | 0 | 0 | 0 | 0 | 1 | 3,537.4 |
|  | 3,537.39 | 0.873 | 0.4348 | 0.3 | 0.7 | 0 | 0 | 0 | 0 | 1 | 3,537.4 |
|  | 3,537.39 | 0.873 | 0.4348 | 0.2 | 0.8 | 0 | 0 | 0 | 0 | 1 | 3,537.4 |
|  | 3,537.39 | 0.873 | 0.4348 | 0.1 | 0.9 | 0 | 0 | 0 | 0 | 1 | 3,537.4 |
|  | 3,537.39 | 0.873 | 0.4348 | 0 | 1 | 0 | 0 | 0 | 0 | 1 | 3,537.4 |
| SCTM-I | 3,537.39 | 0.873 | 0.4348 | 1 | 0 | 0 | 0 | 0 | 1 | 1 | 3,537.4 |
| (1B/4C) | 3,537.39 | 0.873 | 0.4348 | 0.9 | 0 | 0.1 | 0 | 0 | 1 | 1 | 3,537.4 |
|  | 3,537.39 | 0.873 | 0.4348 | 0.8 | 0 | 0.2 | 0 | 0 | 1 | 1 | 3,537.4 |
|  | 3,537.39 | 0.873 | 0.4348 | 0.7 | 0 | 0.3 | 0 | 0 | 1 | 1 | 3,537.4 |
|  | 3,537.39 | 0.873 | 0.4348 | 0.6 | 0 | 0.4 | 0 | 0 | 1 | 1 | 3,537.4 |
|  | 3,537.39 | 0.873 | 0.4348 | 0.5 | 0 | 0.5 | 0 | 0 | 1 | 1 | 3,537.4 |

Supporting Table S1. (continued)

| **Scenario** | **Yield (kg/ha)** | **q** | **p_o_** | **p_w_** | **p_s_** | **p_c_** | **^n^ANY** | **^n^PY** | **^n^OP** | **^n^SF** | **E(Y) (kg/ha)** |
| --- | --- | --- | --- | --- | --- | --- | --- | --- | --- | --- | --- |
|  | 3,537.39 | 0.873 | 0.4348 | 0.4 | 0 | 0.6 | 0 | 0 | 1 | 1 | 3,537.4 |
|  | 3,537.39 | 0.873 | 0.4348 | 0.3 | 0 | 0.7 | 0 | 0 | 1 | 1 | 3,537.4 |
|  | 3,537.39 | 0.873 | 0.4348 | 0.2 | 0 | 0.8 | 0 | 0 | 1 | 1 | 3,537.4 |
|  | 3,537.39 | 0.873 | 0.4348 | 0.1 | 0 | 0.9 | 0 | 0 | 1 | 1 | 3,537.4 |
|  | 3,537.39 | 0.873 | 0.4348 | 0 | 0 | 1 | 0 | 0 | 1 | 1 | 3,537.4 |
| SCTM-I | 3,537.39 | 0.873 | 0.4348 | 1 | 0 | 0 | 0 | 0 | 0 | 1 | 3,537.4 |
| (4C only) | 3,537.39 | 0.873 | 0.4348 | 0.9 | 0 | 0.1 | 0 | 0 | 0 | 1 | 3,537.4 |
|  | 3,537.39 | 0.873 | 0.4348 | 0.8 | 0 | 0.2 | 0 | 0 | 0 | 1 | 3,537.4 |
|  | 3,537.39 | 0.873 | 0.4348 | 0.7 | 0 | 0.3 | 0 | 0 | 0 | 1 | 3,537.4 |
|  | 3,537.39 | 0.873 | 0.4348 | 0.6 | 0 | 0.4 | 0 | 0 | 0 | 1 | 3,537.4 |
|  | 3,537.39 | 0.873 | 0.4348 | 0.5 | 0 | 0.5 | 0 | 0 | 0 | 1 | 3,537.4 |
|  | 3,537.39 | 0.873 | 0.4348 | 0.4 | 0 | 0.6 | 0 | 0 | 0 | 1 | 3,537.4 |
|  | 3,537.39 | 0.873 | 0.4348 | 0.3 | 0 | 0.7 | 0 | 0 | 0 | 1 | 3,537.4 |
|  | 3,537.39 | 0.873 | 0.4348 | 0.2 | 0 | 0.8 | 0 | 0 | 0 | 1 | 3,537.4 |
|  | 3,537.39 | 0.873 | 0.4348 | 0.1 | 0 | 0.9 | 0 | 0 | 0 | 1 | 3,537.4 |
|  | 3,537.39 | 0.873 | 0.4348 | 0 | 0 | 1 | 0 | 0 | 0 | 1 | 3,537.4 |
| RCXN-I | 3,537.39 | 1 | 0.4348 | 1 | 0 | 0 | 0 | 0 | 0 | 0 | 3,537.4 |
|  | 3,537.39 | 1 | 0.4348 | 0.9 | 0.1 | 0 | 0 | 0 | 0 | 0 | 3,537.4 |
|  | 3,537.39 | 1 | 0.4348 | 0.8 | 0.2 | 0 | 0 | 0 | 0 | 0 | 3,537.4 |
|  | 3,537.39 | 1 | 0.4348 | 0.7 | 0.3 | 0 | 0 | 0 | 0 | 0 | 3,537.4 |
|  | 3,537.39 | 1 | 0.4348 | 0.6 | 0.4 | 0 | 0 | 0 | 0 | 0 | 3,537.4 |
|  | 3,537.39 | 1 | 0.4348 | 0.5 | 0.5 | 0 | 0 | 0 | 0 | 0 | 3,537.4 |
|  | 3,537.39 | 1 | 0.4348 | 0.4 | 0.6 | 0 | 0 | 0 | 0 | 0 | 3,537.4 |
|  | 3,537.39 | 1 | 0.4348 | 0.3 | 0.7 | 0 | 0 | 0 | 0 | 0 | 3,537.4 |
|  | 3,537.39 | 1 | 0.4348 | 0.2 | 0.8 | 0 | 0 | 0 | 0 | 0 | 3,537.4 |
|  | 3,537.39 | 1 | 0.4348 | 0.1 | 0.9 | 0 | 0 | 0 | 0 | 0 | 3,537.4 |
|  | 3,537.39 | 1 | 0.4348 | 0 | 1 | 0 | 0 | 0 | 0 | 0 | 3,537.4 |
| RCXN-I | 3,537.39 | 1 | 0.4348 | 1 | 0 | 0 | 0 | 0 | 0 | 0 | 3,537.4 |

Supporting Table S1. (continued)

| **Scenario** | **Yield (kg/ha)** | **q** | **p_o_** | **p_w_** | **p_s_** | **p_c_** | **^n^ANY** | **^n^PY** | **^n^OP** | **^n^SF** | **E(Y) (kg/ha)** |
| --- | --- | --- | --- | --- | --- | --- | --- | --- | --- | --- | --- |
|  | 3,537.39 | 1 | 0.4348 | 0.9 | 0 | 0.1 | 0 | 0 | 0 | 0 | 3,537.4 |
|  | 3,537.39 | 1 | 0.4348 | 0.8 | 0 | 0.2 | 0 | 0 | 0 | 0 | 3,537.4 |
|  | 3,537.39 | 1 | 0.4348 | 0.7 | 0 | 0.3 | 0 | 0 | 0 | 0 | 3,537.4 |
|  | 3,537.39 | 1 | 0.4348 | 0.6 | 0 | 0.4 | 0 | 0 | 0 | 0 | 3,537.4 |
|  | 3,537.39 | 1 | 0.4348 | 0.5 | 0 | 0.5 | 0 | 0 | 0 | 0 | 3,537.4 |
|  | 3,537.39 | 1 | 0.4348 | 0.4 | 0 | 0.6 | 0 | 0 | 0 | 0 | 3,537.4 |
|  | 3,537.39 | 1 | 0.4348 | 0.3 | 0 | 0.7 | 0 | 0 | 0 | 0 | 3,537.4 |
|  | 3,537.39 | 1 | 0.4348 | 0.2 | 0 | 0.8 | 0 | 0 | 0 | 0 | 3,537.4 |
|  | 3,537.39 | 1 | 0.4348 | 0.1 | 0 | 0.9 | 0 | 0 | 0 | 0 | 3,537.4 |
|  | 3,537.39 | 1 | 0.4348 | 0 | 0 | 1 | 0 | 0 | 0 | 0 | 3,537.4 |
| RCXM-I | 3,537.39 | 1 | 0.4348 | 1 | 0 | 0 | 0 | 0 | 0 | 0 | 3,537.4 |
|  | 3,537.39 | 1 | 0.4348 | 0.9 | 0.1 | 0 | 0 | 0 | 0 | 0 | 3,537.4 |
|  | 3,537.39 | 1 | 0.4348 | 0.8 | 0.2 | 0 | 0 | 0 | 0 | 0 | 3,537.4 |
|  | 3,537.39 | 1 | 0.4348 | 0.7 | 0.3 | 0 | 0 | 0 | 0 | 0 | 3,537.4 |
|  | 3,537.39 | 1 | 0.4348 | 0.6 | 0.4 | 0 | 0 | 0 | 0 | 0 | 3,537.4 |
|  | 3,537.39 | 1 | 0.4348 | 0.5 | 0.5 | 0 | 0 | 0 | 0 | 0 | 3,537.4 |
|  | 3,537.39 | 1 | 0.4348 | 0.4 | 0.6 | 0 | 0 | 0 | 0 | 0 | 3,537.4 |
|  | 3,537.39 | 1 | 0.4348 | 0.3 | 0.7 | 0 | 0 | 0 | 0 | 0 | 3,537.4 |
|  | 3,537.39 | 1 | 0.4348 | 0.2 | 0.8 | 0 | 0 | 0 | 0 | 0 | 3,537.4 |
|  | 3,537.39 | 1 | 0.4348 | 0.1 | 0.9 | 0 | 0 | 0 | 0 | 0 | 3,537.4 |
|  | 3,537.39 | 1 | 0.4348 | 0 | 1 | 0 | 0 | 0 | 0 | 0 | 3,537.4 |
| RCXM-I | 3,537.39 | 1 | 0.4348 | 1 | 0 | 0 | 0 | 0 | 0 | 0 | 3,537.4 |
|  | 3,537.39 | 1 | 0.4348 | 0.9 | 0 | 0.1 | 0 | 0 | 0 | 0 | 3,537.4 |
|  | 3,537.39 | 1 | 0.4348 | 0.8 | 0 | 0.2 | 0 | 0 | 0 | 0 | 3,537.4 |
|  | 3,537.39 | 1 | 0.4348 | 0.7 | 0 | 0.3 | 0 | 0 | 0 | 0 | 3,537.4 |
|  | 3,537.39 | 1 | 0.4348 | 0.6 | 0 | 0.4 | 0 | 0 | 0 | 0 | 3,537.4 |
|  | 3,537.39 | 1 | 0.4348 | 0.5 | 0 | 0.5 | 0 | 0 | 0 | 0 | 3,537.4 |
|  | 3,537.39 | 1 | 0.4348 | 0.4 | 0 | 0.6 | 0 | 0 | 0 | 0 | 3,537.4 |

Supporting Table S1. (continued)

| **Scenario** | **Yield (kg/ha)** | **q** | **p_o_** | **p_w_** | **p_s_** | **p_c_** | **^n^ANY** | **^n^PY** | **^n^OP** | **^n^SF** | **E(Y) (kg/ha)** |
| --- | --- | --- | --- | --- | --- | --- | --- | --- | --- | --- | --- |
|  | 3,537.39 | 1 | 0.4348 | 0.3 | 0 | 0.7 | 0 | 0 | 0 | 0 | 3,537.4 |
|  | 3,537.39 | 1 | 0.4348 | 0.2 | 0 | 0.8 | 0 | 0 | 0 | 0 | 3,537.4 |
|  | 3,537.39 | 1 | 0.4348 | 0.1 | 0 | 0.9 | 0 | 0 | 0 | 0 | 3,537.4 |
|  | 3,537.39 | 1 | 0.4348 | 0 | 0 | 1 | 0 | 0 | 0 | 0 | 3,537.4 |

1. Refer to the Materials and Methods section of the manuscript for a description of scenario abbreviations and terms. [↑](#footnote-ref-1)
2. The 10-year average yield in Iowa. [↑](#footnote-ref-2)
3. The proportion of yield expected in Iowa, based on observed yield loss when an outbreak occurs. [↑](#footnote-ref-3)
4. The probability of an outbreak occurring (p_o_). Within an outbreak, aphids could be wild-type (p_w_), pyrethroid-resistant (p_s_), or cross-resistant to organophosphates (p_c_). [↑](#footnote-ref-4)
5. The insecticide applied in the scenario. All “0” indicates no insecticide is applied. A “1” in any column indicates that particular insecticide is applied. ^n^ANY refers to any insecticide application, ^n^PY is a pyrethroid, ^n^OP is an organophosphate, and ^n^SF is sulfoxaflor. [↑](#footnote-ref-5)
6. Expected yield calculated using Equation 1 and these parameter values. [↑](#footnote-ref-6)
