# Supplementary material for: Developing a decision‐making framework for insect pest management: a case study using Aphis glycines (Hemiptera: Aphididae)
Source: Pest Manag Sci. 2020 Oct 1;77(2):886–94. doi: 10.1002/ps.6093 (PMC7821323; doi:10.1002/ps.6093)
Supplement: Supplementary file 3 — Supporting Table S2. Expected net revenue (E[R]) parameters and estimates for all soybean aphid management scenarios analyzed. [file PS-77-886-s003.docx]

Supporting Table S2. Expected net revenue (*E*[R]) parameters and estimates for all soybean aphid management scenarios analyzed.

| **Scenario^[[1]](#footnote-1)^** | **p_o_^[[2]](#footnote-2)^** | **p_w_^2^** | **p_s_^2^** | **p_c_^2^** | ***E*(Y) (kg/ha)^[[3]](#footnote-3)^** | ***E*(C) ($/ha)^[[4]](#footnote-4)^** | ***E*(P) ($/kg)^[[5]](#footnote-5)^** | ***E*(R) ($/ha)^[[6]](#footnote-6)^** |
| --- | --- | --- | --- | --- | --- | --- | --- | --- |
| SHXN-W | 0 | 1 | 0 | 0 | 3,537.4 | 244.8 | 0.33 | 922.51 |
|  | 0.1 | 1 | 0 | 0 | 3,492.5 | 244.8 | 0.33 | 907.69 |
|  | 0.2 | 1 | 0 | 0 | 3,447.5 | 244.8 | 0.33 | 892.86 |
|  | 0.3 | 1 | 0 | 0 | 3,402.6 | 244.8 | 0.33 | 878.04 |
|  | 0.4 | 1 | 0 | 0 | 3,357.7 | 244.8 | 0.33 | 863.21 |
|  | 0.5 | 1 | 0 | 0 | 3,312.8 | 244.8 | 0.33 | 848.39 |
|  | 0.6 | 1 | 0 | 0 | 3,267.8 | 244.8 | 0.33 | 833.56 |
|  | 0.7 | 1 | 0 | 0 | 3,222.9 | 244.8 | 0.33 | 818.74 |
|  | 0.8 | 1 | 0 | 0 | 3,178.0 | 244.8 | 0.33 | 803.91 |
|  | 0.9 | 1 | 0 | 0 | 3,133.1 | 244.8 | 0.33 | 789.08 |
|  | 1 | 1 | 0 | 0 | 3,088.1 | 244.8 | 0.33 | 774.26 |
| SHPN-W | 0 | 1 | 0 | 0 | 3,537.4 | 287.7 | 0.33 | 879.62 |
|  | 0.1 | 1 | 0 | 0 | 3,537.4 | 287.7 | 0.33 | 879.62 |
|  | 0.2 | 1 | 0 | 0 | 3,537.4 | 287.7 | 0.33 | 879.62 |
|  | 0.3 | 1 | 0 | 0 | 3,537.4 | 287.7 | 0.33 | 879.62 |
|  | 0.4 | 1 | 0 | 0 | 3,537.4 | 287.7 | 0.33 | 879.62 |
|  | 0.5 | 1 | 0 | 0 | 3,537.4 | 287.7 | 0.33 | 879.62 |
|  | 0.6 | 1 | 0 | 0 | 3,537.4 | 287.7 | 0.33 | 879.62 |
|  | 0.7 | 1 | 0 | 0 | 3,537.4 | 287.7 | 0.33 | 879.62 |

Supporting Table S2. (continued)

| **Scenario** | **p_o_** | **p_w_** | **p_s_** | **p_c_** | ***E*(Y) (kg/ha)** | ***E*(C) ($/ha)** | ***E*(P) ($/kg)** | ***E*(R) ($/ha)** |
| --- | --- | --- | --- | --- | --- | --- | --- | --- |
|  | 0.8 | 1 | 0 | 0 | 3,537.4 | 287.7 | 0.33 | 879.62 |
|  | 0.9 | 1 | 0 | 0 | 3,537.4 | 287.7 | 0.33 | 879.62 |
|  | 1 | 1 | 0 | 0 | 3,537.4 | 287.7 | 0.33 | 879.62 |
| SHPE-W | 0 | 1 | 0 | 0 | 3,537.4 | 298.2 | 0.33 | 869.11 |
|  | 0.1 | 1 | 0 | 0 | 3,537.4 | 298.2 | 0.33 | 869.11 |
|  | 0.2 | 1 | 0 | 0 | 3,537.4 | 298.2 | 0.33 | 869.11 |
|  | 0.3 | 1 | 0 | 0 | 3,537.4 | 298.2 | 0.33 | 869.11 |
|  | 0.4 | 1 | 0 | 0 | 3,537.4 | 298.2 | 0.33 | 869.11 |
|  | 0.5 | 1 | 0 | 0 | 3,537.4 | 298.2 | 0.33 | 869.11 |
|  | 0.6 | 1 | 0 | 0 | 3,537.4 | 298.2 | 0.33 | 869.11 |
|  | 0.7 | 1 | 0 | 0 | 3,537.4 | 298.2 | 0.33 | 869.11 |
|  | 0.8 | 1 | 0 | 0 | 3,537.4 | 298.2 | 0.33 | 869.11 |
|  | 0.9 | 1 | 0 | 0 | 3,537.4 | 298.2 | 0.33 | 869.11 |
|  | 1 | 1 | 0 | 0 | 3,537.4 | 298.2 | 0.33 | 869.11 |
| SHTM-W | 0 | 1 | 0 | 0 | 3,537.4 | 361.2 | 0.33 | 859.50 |
|  | 0.1 | 1 | 0 | 0 | 3,537.4 | 361.2 | 0.33 | 854.16 |
|  | 0.2 | 1 | 0 | 0 | 3,537.4 | 361.2 | 0.33 | 848.82 |
|  | 0.3 | 1 | 0 | 0 | 3,537.4 | 361.2 | 0.33 | 843.48 |
|  | 0.4 | 1 | 0 | 0 | 3,537.4 | 361.2 | 0.33 | 838.14 |
|  | 0.5 | 1 | 0 | 0 | 3,537.4 | 361.2 | 0.33 | 832.80 |
|  | 0.6 | 1 | 0 | 0 | 3,537.4 | 361.2 | 0.33 | 827.46 |
|  | 0.7 | 1 | 0 | 0 | 3,537.4 | 361.2 | 0.33 | 822.12 |
|  | 0.8 | 1 | 0 | 0 | 3,537.4 | 361.2 | 0.33 | 816.78 |
|  | 0.9 | 1 | 0 | 0 | 3,537.4 | 361.2 | 0.33 | 811.44 |
|  | 1 | 1 | 0 | 0 | 3,537.4 | 361.2 | 0.33 | 806.10 |
| SCXN-W | 0 | 1 | 0 | 0 | 3,537.4 | 236.3 | 0.33 | 931.09 |
|  | 0.1 | 1 | 0 | 0 | 3,492.5 | 236.3 | 0.33 | 916.26 |
|  | 0.2 | 1 | 0 | 0 | 3,447.5 | 236.3 | 0.33 | 901.44 |

Supporting Table S2. (continued)

| **Scenario** | **p_o_** | **p_w_** | **p_s_** | **p_c_** | ***E*(Y) (kg/ha)** | ***E*(C) ($/ha)** | ***E*(P) ($/kg)** | ***E*(R) ($/ha)** |
| --- | --- | --- | --- | --- | --- | --- | --- | --- |
|  | 0.3 | 1 | 0 | 0 | 3,402.6 | 236.3 | 0.33 | 886.61 |
|  | 0.4 | 1 | 0 | 0 | 3,357.7 | 236.3 | 0.33 | 871.79 |
|  | 0.5 | 1 | 0 | 0 | 3,312.8 | 236.3 | 0.33 | 856.96 |
|  | 0.6 | 1 | 0 | 0 | 3,267.8 | 236.3 | 0.33 | 842.13 |
|  | 0.7 | 1 | 0 | 0 | 3,222.9 | 236.3 | 0.33 | 827.31 |
|  | 0.8 | 1 | 0 | 0 | 3,178.0 | 236.3 | 0.33 | 812.48 |
|  | 0.9 | 1 | 0 | 0 | 3,133.1 | 236.3 | 0.33 | 797.66 |
|  | 1 | 1 | 0 | 0 | 3,088.1 | 236.3 | 0.33 | 782.83 |
| SCPN-W | 0 | 1 | 0 | 0 | 3,537.4 | 279.1 | 0.33 | 888.19 |
|  | 0.1 | 1 | 0 | 0 | 3,537.4 | 279.1 | 0.33 | 888.19 |
|  | 0.2 | 1 | 0 | 0 | 3,537.4 | 279.1 | 0.33 | 888.19 |
|  | 0.3 | 1 | 0 | 0 | 3,537.4 | 279.1 | 0.33 | 888.19 |
|  | 0.4 | 1 | 0 | 0 | 3,537.4 | 279.1 | 0.33 | 888.19 |
|  | 0.5 | 1 | 0 | 0 | 3,537.4 | 279.1 | 0.33 | 888.19 |
|  | 0.6 | 1 | 0 | 0 | 3,537.4 | 279.1 | 0.33 | 888.19 |
|  | 0.7 | 1 | 0 | 0 | 3,537.4 | 279.1 | 0.33 | 888.19 |
|  | 0.8 | 1 | 0 | 0 | 3,537.4 | 279.1 | 0.33 | 888.19 |
|  | 0.9 | 1 | 0 | 0 | 3,537.4 | 279.1 | 0.33 | 888.19 |
|  | 1 | 1 | 0 | 0 | 3,537.4 | 279.1 | 0.33 | 888.19 |
| SCPE-W | 0 | 1 | 0 | 0 | 3,537.4 | 289.7 | 0.33 | 877.69 |
|  | 0.1 | 1 | 0 | 0 | 3,537.4 | 289.7 | 0.33 | 877.69 |
|  | 0.2 | 1 | 0 | 0 | 3,537.4 | 289.7 | 0.33 | 877.69 |
|  | 0.3 | 1 | 0 | 0 | 3,537.4 | 289.7 | 0.33 | 877.69 |
|  | 0.4 | 1 | 0 | 0 | 3,537.4 | 289.7 | 0.33 | 877.69 |
|  | 0.5 | 1 | 0 | 0 | 3,537.4 | 289.7 | 0.33 | 877.69 |
|  | 0.6 | 1 | 0 | 0 | 3,537.4 | 289.7 | 0.33 | 877.69 |
|  | 0.7 | 1 | 0 | 0 | 3,537.4 | 289.7 | 0.33 | 877.69 |
|  | 0.8 | 1 | 0 | 0 | 3,537.4 | 289.7 | 0.33 | 877.69 |

Supporting Table S2. (continued)

| **Scenario** | **p_o_** | **p_w_** | **p_s_** | **p_c_** | ***E*(Y) (kg/ha)** | ***E*(C) ($/ha)** | ***E*(P) ($/kg)** | ***E*(R) ($/ha)** |
| --- | --- | --- | --- | --- | --- | --- | --- | --- |
|  | 0.9 | 1 | 0 | 0 | 3,537.4 | 289.7 | 0.33 | 877.69 |
|  | 1 | 1 | 0 | 0 | 3,537.4 | 289.7 | 0.33 | 877.69 |
| SCTM-W | 0 | 1 | 0 | 0 | 3,537.4 | 352.7 | 0.33 | 868.08 |
|  | 0.1 | 1 | 0 | 0 | 3,537.4 | 352.7 | 0.33 | 862.74 |
|  | 0.2 | 1 | 0 | 0 | 3,537.4 | 352.7 | 0.33 | 857.40 |
|  | 0.3 | 1 | 0 | 0 | 3,537.4 | 352.7 | 0.33 | 852.06 |
|  | 0.4 | 1 | 0 | 0 | 3,537.4 | 352.7 | 0.33 | 846.72 |
|  | 0.5 | 1 | 0 | 0 | 3,537.4 | 352.7 | 0.33 | 841.38 |
|  | 0.6 | 1 | 0 | 0 | 3,537.4 | 352.7 | 0.33 | 836.04 |
|  | 0.7 | 1 | 0 | 0 | 3,537.4 | 352.7 | 0.33 | 830.70 |
|  | 0.8 | 1 | 0 | 0 | 3,537.4 | 352.7 | 0.33 | 825.36 |
|  | 0.9 | 1 | 0 | 0 | 3,537.4 | 352.7 | 0.33 | 820.02 |
|  | 1 | 1 | 0 | 0 | 3,537.4 | 352.7 | 0.33 | 814.68 |
| RCXN-W | 0 | 1 | 0 | 0 | 3,537.4 | 233.0 | 0.33 | 934.37 |
|  | 0.1 | 1 | 0 | 0 | 3,537.4 | 233.0 | 0.33 | 934.37 |
|  | 0.2 | 1 | 0 | 0 | 3,537.4 | 233.0 | 0.33 | 934.37 |
|  | 0.3 | 1 | 0 | 0 | 3,537.4 | 233.0 | 0.33 | 934.37 |
|  | 0.4 | 1 | 0 | 0 | 3,537.4 | 233.0 | 0.33 | 934.37 |
|  | 0.5 | 1 | 0 | 0 | 3,537.4 | 233.0 | 0.33 | 934.37 |
|  | 0.6 | 1 | 0 | 0 | 3,537.4 | 233.0 | 0.33 | 934.37 |
|  | 0.7 | 1 | 0 | 0 | 3,537.4 | 233.0 | 0.33 | 934.37 |
|  | 0.8 | 1 | 0 | 0 | 3,537.4 | 233.0 | 0.33 | 934.37 |
|  | 0.9 | 1 | 0 | 0 | 3,537.4 | 233.0 | 0.33 | 934.37 |
|  | 1 | 1 | 0 | 0 | 3,537.4 | 233.0 | 0.33 | 934.37 |
| RCXM-W | 0 | 1 | 0 | 0 | 3,537.4 | 296.0 | 0.33 | 871.36 |
|  | 0.1 | 1 | 0 | 0 | 3,537.4 | 296.0 | 0.33 | 871.36 |
|  | 0.2 | 1 | 0 | 0 | 3,537.4 | 296.0 | 0.33 | 871.36 |
|  | 0.3 | 1 | 0 | 0 | 3,537.4 | 296.0 | 0.33 | 871.36 |

Supporting Table S2. (continued)

| **Scenario** | **p_o_** | **p_w_** | **p_s_** | **p_c_** | ***E*(Y) (kg/ha)** | ***E*(C) ($/ha)** | ***E*(P) ($/kg)** | ***E*(R) ($/ha)** |
| --- | --- | --- | --- | --- | --- | --- | --- | --- |
|  | 0.4 | 1 | 0 | 0 | 3,537.4 | 296.0 | 0.33 | 871.36 |
|  | 0.5 | 1 | 0 | 0 | 3,537.4 | 296.0 | 0.33 | 871.36 |
|  | 0.6 | 1 | 0 | 0 | 3,537.4 | 296.0 | 0.33 | 871.36 |
|  | 0.7 | 1 | 0 | 0 | 3,537.4 | 296.0 | 0.33 | 871.36 |
|  | 0.8 | 1 | 0 | 0 | 3,537.4 | 296.0 | 0.33 | 871.36 |
|  | 0.9 | 1 | 0 | 0 | 3,537.4 | 296.0 | 0.33 | 871.36 |
|  | 1 | 1 | 0 | 0 | 3,537.4 | 296.0 | 0.33 | 871.36 |
| SHXN-I | 0.4348 | 1 | 0 | 0 | 3,342.1 | 244.8 | 0.33 | 858.05 |
|  | 0.4348 | 0.9 | 0.1 | 0 | 3,342.1 | 244.8 | 0.33 | 858.05 |
|  | 0.4348 | 0.8 | 0.2 | 0 | 3,342.1 | 244.8 | 0.33 | 858.05 |
|  | 0.4348 | 0.7 | 0.3 | 0 | 3,342.1 | 244.8 | 0.33 | 858.05 |
|  | 0.4348 | 0.6 | 0.4 | 0 | 3,342.1 | 244.8 | 0.33 | 858.05 |
|  | 0.4348 | 0.5 | 0.5 | 0 | 3,342.1 | 244.8 | 0.33 | 858.05 |
|  | 0.4348 | 0.4 | 0.6 | 0 | 3,342.1 | 244.8 | 0.33 | 858.05 |
|  | 0.4348 | 0.3 | 0.7 | 0 | 3,342.1 | 244.8 | 0.33 | 858.05 |
|  | 0.4348 | 0.2 | 0.8 | 0 | 3,342.1 | 244.8 | 0.33 | 858.05 |
|  | 0.4348 | 0.1 | 0.9 | 0 | 3,342.1 | 244.8 | 0.33 | 858.05 |
|  | 0.4348 | 0 | 1 | 0 | 3,342.1 | 244.8 | 0.33 | 858.05 |
| SHPN-I | 0.4348 | 1 | 0 | 0 | 3,537.4 | 278.7 | 0.33 | 888.66 |
|  | 0.4348 | 0.9 | 0.1 | 0 | 3,517.9 | 278.7 | 0.33 | 882.21 |
|  | 0.4348 | 0.8 | 0.2 | 0 | 3,498.3 | 278.7 | 0.33 | 875.77 |
|  | 0.4348 | 0.7 | 0.3 | 0 | 3,478.8 | 278.7 | 0.33 | 869.32 |
|  | 0.4348 | 0.6 | 0.4 | 0 | 3,459.3 | 278.7 | 0.33 | 862.87 |
|  | 0.4348 | 0.5 | 0.5 | 0 | 3,439.7 | 278.7 | 0.33 | 856.43 |
|  | 0.4348 | 0.4 | 0.6 | 0 | 3,420.2 | 278.7 | 0.33 | 849.98 |
|  | 0.4348 | 0.3 | 0.7 | 0 | 3,400.7 | 278.7 | 0.33 | 843.54 |
|  | 0.4348 | 0.2 | 0.8 | 0 | 3,381.1 | 278.7 | 0.33 | 837.09 |
|  | 0.4348 | 0.1 | 0.9 | 0 | 3,361.6 | 278.7 | 0.33 | 830.64 |

Supporting Table S2. (continued)

| **Scenario** | **p_o_** | **p_w_** | **p_s_** | **p_c_** | ***E*(Y) (kg/ha)** | ***E*(C) ($/ha)** | ***E*(P) ($/kg)** | ***E*(R) ($/ha)** |
| --- | --- | --- | --- | --- | --- | --- | --- | --- |
|  | 0.4348 | 0 | 1 | 0 | 3,342.1 | 278.7 | 0.33 | 824.20 |
| SHPN-I | 0.4348 | 1 | 0 | 0 | 3,537.4 | 286.8 | 0.33 | 880.50 |
| (1B only) | 0.4348 | 0.9 | 0.1 | 0 | 3,537.4 | 286.8 | 0.33 | 880.50 |
|  | 0.4348 | 0.8 | 0.2 | 0 | 3,537.4 | 286.8 | 0.33 | 880.50 |
|  | 0.4348 | 0.7 | 0.3 | 0 | 3,537.4 | 286.8 | 0.33 | 880.50 |
|  | 0.4348 | 0.6 | 0.4 | 0 | 3,537.4 | 286.8 | 0.33 | 880.50 |
|  | 0.4348 | 0.5 | 0.5 | 0 | 3,537.4 | 286.8 | 0.33 | 880.50 |
|  | 0.4348 | 0.4 | 0.6 | 0 | 3,537.4 | 286.8 | 0.33 | 880.50 |
|  | 0.4348 | 0.3 | 0.7 | 0 | 3,537.4 | 286.8 | 0.33 | 880.50 |
|  | 0.4348 | 0.2 | 0.8 | 0 | 3,537.4 | 286.8 | 0.33 | 880.50 |
|  | 0.4348 | 0.1 | 0.9 | 0 | 3,537.4 | 286.8 | 0.33 | 880.50 |
|  | 0.4348 | 0 | 1 | 0 | 3,537.4 | 286.8 | 0.33 | 880.50 |
| SHPN-I | 0.4348 | 1 | 0 | 0 | 3,537.4 | 326.6 | 0.33 | 840.72 |
| (4C only) | 0.4348 | 0.9 | 0.1 | 0 | 3,537.4 | 326.6 | 0.33 | 840.72 |
|  | 0.4348 | 0.8 | 0.2 | 0 | 3,537.4 | 326.6 | 0.33 | 840.72 |
|  | 0.4348 | 0.7 | 0.3 | 0 | 3,537.4 | 326.6 | 0.33 | 840.72 |
|  | 0.4348 | 0.6 | 0.4 | 0 | 3,537.4 | 326.6 | 0.33 | 840.72 |
|  | 0.4348 | 0.5 | 0.5 | 0 | 3,537.4 | 326.6 | 0.33 | 840.72 |
|  | 0.4348 | 0.4 | 0.6 | 0 | 3,537.4 | 326.6 | 0.33 | 840.72 |
|  | 0.4348 | 0.3 | 0.7 | 0 | 3,537.4 | 326.6 | 0.33 | 840.72 |
|  | 0.4348 | 0.2 | 0.8 | 0 | 3,537.4 | 326.6 | 0.33 | 840.72 |
|  | 0.4348 | 0.1 | 0.9 | 0 | 3,537.4 | 326.6 | 0.33 | 840.72 |
|  | 0.4348 | 0 | 1 | 0 | 3,537.4 | 326.6 | 0.33 | 840.72 |
| SHPE-I | 0.4348 | 1 | 0 | 0 | 3,537.4 | 341.7 | 0.33 | 878.16 |
| (3A/1B) | 0.4348 | 0.9 | 0.1 | 0 | 3,537.4 | 341.7 | 0.33 | 872.91 |
|  | 0.4348 | 0.8 | 0.2 | 0 | 3,537.4 | 341.7 | 0.33 | 867.66 |
|  | 0.4348 | 0.7 | 0.3 | 0 | 3,537.4 | 341.7 | 0.33 | 862.40 |
|  | 0.4348 | 0.6 | 0.4 | 0 | 3,537.4 | 341.7 | 0.33 | 857.15 |

Supporting Table S2. (continued)

| **Scenario** | **p_o_** | **p_w_** | **p_s_** | **p_c_** | ***E*(Y) (kg/ha)** | ***E*(C) ($/ha)** | ***E*(P) ($/kg)** | ***E*(R) ($/ha)** |
| --- | --- | --- | --- | --- | --- | --- | --- | --- |
|  | 0.4348 | 0.5 | 0.5 | 0 | 3,537.4 | 341.7 | 0.33 | 851.90 |
|  | 0.4348 | 0.4 | 0.6 | 0 | 3,537.4 | 341.7 | 0.33 | 846.65 |
|  | 0.4348 | 0.3 | 0.7 | 0 | 3,537.4 | 341.7 | 0.33 | 841.40 |
|  | 0.4348 | 0.2 | 0.8 | 0 | 3,537.4 | 341.7 | 0.33 | 836.15 |
|  | 0.4348 | 0.1 | 0.9 | 0 | 3,537.4 | 341.7 | 0.33 | 830.90 |
|  | 0.4348 | 0 | 1 | 0 | 3,537.4 | 341.7 | 0.33 | 825.65 |
| SHPE-I | 0.4348 | 1 | 0 | 0 | 3,537.4 | 337.1 | 0.33 | 830.22 |
| (4C only) | 0.4348 | 0.9 | 0.1 | 0 | 3,537.4 | 337.1 | 0.33 | 830.22 |
|  | 0.4348 | 0.8 | 0.2 | 0 | 3,537.4 | 337.1 | 0.33 | 830.22 |
|  | 0.4348 | 0.7 | 0.3 | 0 | 3,537.4 | 337.1 | 0.33 | 830.22 |
|  | 0.4348 | 0.6 | 0.4 | 0 | 3,537.4 | 337.1 | 0.33 | 830.22 |
|  | 0.4348 | 0.5 | 0.5 | 0 | 3,537.4 | 337.1 | 0.33 | 830.22 |
|  | 0.4348 | 0.4 | 0.6 | 0 | 3,537.4 | 337.1 | 0.33 | 830.22 |
|  | 0.4348 | 0.3 | 0.7 | 0 | 3,537.4 | 337.1 | 0.33 | 830.22 |
|  | 0.4348 | 0.2 | 0.8 | 0 | 3,537.4 | 337.1 | 0.33 | 830.22 |
|  | 0.4348 | 0.1 | 0.9 | 0 | 3,537.4 | 337.1 | 0.33 | 830.22 |
|  | 0.4348 | 0 | 1 | 0 | 3,537.4 | 337.1 | 0.33 | 830.22 |
| SHPN-I | 0.4348 | 1 | 0 | 0 | 3,537.4 | 326.6 | 0.33 | 840.72 |
| (4C only) | 0.4348 | 0.9 | 0 | 0.1 | 3,537.4 | 326.6 | 0.33 | 840.72 |
|  | 0.4348 | 0.8 | 0 | 0.2 | 3,537.4 | 326.6 | 0.33 | 840.72 |
|  | 0.4348 | 0.7 | 0 | 0.3 | 3,537.4 | 326.6 | 0.33 | 840.72 |
|  | 0.4348 | 0.6 | 0 | 0.4 | 3,537.4 | 326.6 | 0.33 | 840.72 |
|  | 0.4348 | 0.5 | 0 | 0.5 | 3,537.4 | 326.6 | 0.33 | 840.72 |
|  | 0.4348 | 0.4 | 0 | 0.6 | 3,537.4 | 326.6 | 0.33 | 840.72 |
|  | 0.4348 | 0.3 | 0 | 0.7 | 3,537.4 | 326.6 | 0.33 | 840.72 |
|  | 0.4348 | 0.2 | 0 | 0.8 | 3,537.4 | 326.6 | 0.33 | 840.72 |
|  | 0.4348 | 0.1 | 0 | 0.9 | 3,537.4 | 326.6 | 0.33 | 840.72 |
|  | 0.4348 | 0 | 0 | 1 | 3,537.4 | 326.6 | 0.33 | 840.72 |

Supporting Table S2. (continued)

| **Scenario** | **p_o_** | **p_w_** | **p_s_** | **p_c_** | ***E*(Y) (kg/ha)** | ***E*(C) ($/ha)** | ***E*(P) ($/kg)** | ***E*(R) ($/ha)** |
| --- | --- | --- | --- | --- | --- | --- | --- | --- |
| SHPE-I | 0.4348 | 1 | 0 | 0 | 3,537.4 | 389.6 | 0.33 | 870.00 |
| (1B/4C) | 0.4348 | 0.9 | 0 | 0.1 | 3,537.4 | 389.6 | 0.33 | 860.77 |
|  | 0.4348 | 0.8 | 0 | 0.2 | 3,537.4 | 389.6 | 0.33 | 851.54 |
|  | 0.4348 | 0.7 | 0 | 0.3 | 3,537.4 | 389.6 | 0.33 | 842.32 |
|  | 0.4348 | 0.6 | 0 | 0.4 | 3,537.4 | 389.6 | 0.33 | 833.09 |
|  | 0.4348 | 0.5 | 0 | 0.5 | 3,537.4 | 389.6 | 0.33 | 823.86 |
|  | 0.4348 | 0.4 | 0 | 0.6 | 3,537.4 | 389.6 | 0.33 | 814.63 |
|  | 0.4348 | 0.3 | 0 | 0.7 | 3,537.4 | 389.6 | 0.33 | 805.40 |
|  | 0.4348 | 0.2 | 0 | 0.8 | 3,537.4 | 389.6 | 0.33 | 796.17 |
|  | 0.4348 | 0.1 | 0 | 0.9 | 3,537.4 | 389.6 | 0.33 | 786.94 |
|  | 0.4348 | 0 | 0 | 1 | 3,537.4 | 389.6 | 0.33 | 777.71 |
| SHTM-I | 0.4348 | 1 | 0 | 0 | 3,537.4 | 404.7 | 0.33 | 815.15 |
| (3A/1B) | 0.4348 | 0.9 | 0.1 | 0 | 3,537.4 | 404.7 | 0.33 | 809.90 |
|  | 0.4348 | 0.8 | 0.2 | 0 | 3,537.4 | 404.7 | 0.33 | 804.64 |
|  | 0.4348 | 0.7 | 0.3 | 0 | 3,537.4 | 404.7 | 0.33 | 799.39 |
|  | 0.4348 | 0.6 | 0.4 | 0 | 3,537.4 | 404.7 | 0.33 | 794.14 |
|  | 0.4348 | 0.5 | 0.5 | 0 | 3,537.4 | 404.7 | 0.33 | 788.89 |
|  | 0.4348 | 0.4 | 0.6 | 0 | 3,537.4 | 404.7 | 0.33 | 783.64 |
|  | 0.4348 | 0.3 | 0.7 | 0 | 3,537.4 | 404.7 | 0.33 | 778.39 |
|  | 0.4348 | 0.2 | 0.8 | 0 | 3,537.4 | 404.7 | 0.33 | 773.14 |
|  | 0.4348 | 0.1 | 0.9 | 0 | 3,537.4 | 404.7 | 0.33 | 767.89 |
|  | 0.4348 | 0 | 1 | 0 | 3,537.4 | 404.7 | 0.33 | 762.64 |
| SHTM-I | 0.4348 | 1 | 0 | 0 | 3,537.4 | 360.3 | 0.33 | 806.99 |
| (1B only) | 0.4348 | 0.9 | 0.1 | 0 | 3,537.4 | 360.3 | 0.33 | 806.99 |
|  | 0.4348 | 0.8 | 0.2 | 0 | 3,537.4 | 360.3 | 0.33 | 806.99 |
|  | 0.4348 | 0.7 | 0.3 | 0 | 3,537.4 | 360.3 | 0.33 | 806.99 |
|  | 0.4348 | 0.6 | 0.4 | 0 | 3,537.4 | 360.3 | 0.33 | 806.99 |
|  | 0.4348 | 0.5 | 0.5 | 0 | 3,537.4 | 360.3 | 0.33 | 806.99 |

Supporting Table S2. (continued)

| **Scenario** | **p_o_** | **p_w_** | **p_s_** | **p_c_** | ***E*(Y) (kg/ha)** | ***E*(C) ($/ha)** | ***E*(P) ($/kg)** | ***E*(R) ($/ha)** |
| --- | --- | --- | --- | --- | --- | --- | --- | --- |
|  | 0.4348 | 0.4 | 0.6 | 0 | 3,537.4 | 360.3 | 0.33 | 806.99 |
|  | 0.4348 | 0.3 | 0.7 | 0 | 3,537.4 | 360.3 | 0.33 | 806.99 |
|  | 0.4348 | 0.2 | 0.8 | 0 | 3,537.4 | 360.3 | 0.33 | 806.99 |
|  | 0.4348 | 0.1 | 0.9 | 0 | 3,537.4 | 360.3 | 0.33 | 806.99 |
|  | 0.4348 | 0 | 1 | 0 | 3,537.4 | 360.3 | 0.33 | 806.99 |
| SHTM-I | 0.4348 | 1 | 0 | 0 | 3,537.4 | 400.1 | 0.33 | 767.21 |
| (4C only) | 0.4348 | 0.9 | 0.1 | 0 | 3,537.4 | 400.1 | 0.33 | 767.21 |
|  | 0.4348 | 0.8 | 0.2 | 0 | 3,537.4 | 400.1 | 0.33 | 767.21 |
|  | 0.4348 | 0.7 | 0.3 | 0 | 3,537.4 | 400.1 | 0.33 | 767.21 |
|  | 0.4348 | 0.6 | 0.4 | 0 | 3,537.4 | 400.1 | 0.33 | 767.21 |
|  | 0.4348 | 0.5 | 0.5 | 0 | 3,537.4 | 400.1 | 0.33 | 767.21 |
|  | 0.4348 | 0.4 | 0.6 | 0 | 3,537.4 | 400.1 | 0.33 | 767.21 |
|  | 0.4348 | 0.3 | 0.7 | 0 | 3,537.4 | 400.1 | 0.33 | 767.21 |
|  | 0.4348 | 0.2 | 0.8 | 0 | 3,537.4 | 400.1 | 0.33 | 767.21 |
|  | 0.4348 | 0.1 | 0.9 | 0 | 3,537.4 | 400.1 | 0.33 | 767.21 |
|  | 0.4348 | 0 | 1 | 0 | 3,537.4 | 400.1 | 0.33 | 767.21 |
| SHTM-I | 0.4348 | 1 | 0 | 0 | 3,537.4 | 452.6 | 0.33 | 806.99 |
| (1B/4C) | 0.4348 | 0.9 | 0 | 0.1 | 3,537.4 | 452.6 | 0.33 | 797.76 |
|  | 0.4348 | 0.8 | 0 | 0.2 | 3,537.4 | 452.6 | 0.33 | 788.53 |
|  | 0.4348 | 0.7 | 0 | 0.3 | 3,537.4 | 452.6 | 0.33 | 779.30 |
|  | 0.4348 | 0.6 | 0 | 0.4 | 3,537.4 | 452.6 | 0.33 | 770.08 |
|  | 0.4348 | 0.5 | 0 | 0.5 | 3,537.4 | 452.6 | 0.33 | 760.85 |
|  | 0.4348 | 0.4 | 0 | 0.6 | 3,537.4 | 452.6 | 0.33 | 751.62 |
|  | 0.4348 | 0.3 | 0 | 0.7 | 3,537.4 | 452.6 | 0.33 | 742.39 |
|  | 0.4348 | 0.2 | 0 | 0.8 | 3,537.4 | 452.6 | 0.33 | 733.16 |
|  | 0.4348 | 0.1 | 0 | 0.9 | 3,537.4 | 452.6 | 0.33 | 723.93 |
|  | 0.4348 | 0 | 0 | 1 | 3,537.4 | 452.6 | 0.33 | 714.70 |

Supporting Table S2. (continued)

| **Scenario** | **p_o_** | **p_w_** | **p_s_** | **p_c_** | ***E*(Y) (kg/ha)** | ***E*(C) ($/ha)** | ***E*(P) ($/kg)** | ***E*(R) ($/ha)** |
| --- | --- | --- | --- | --- | --- | --- | --- | --- |
| SHTM-I | 0.4348 | 1 | 0 | 0 | 3,537.4 | 400.1 | 0.33 | 767.21 |
| (4C only) | 0.4348 | 0.9 | 0 | 0.1 | 3,537.4 | 400.1 | 0.33 | 767.21 |
|  | 0.4348 | 0.8 | 0 | 0.2 | 3,537.4 | 400.1 | 0.33 | 767.21 |
|  | 0.4348 | 0.7 | 0 | 0.3 | 3,537.4 | 400.1 | 0.33 | 767.21 |
|  | 0.4348 | 0.6 | 0 | 0.4 | 3,537.4 | 400.1 | 0.33 | 767.21 |
|  | 0.4348 | 0.5 | 0 | 0.5 | 3,537.4 | 400.1 | 0.33 | 767.21 |
|  | 0.4348 | 0.4 | 0 | 0.6 | 3,537.4 | 400.1 | 0.33 | 767.21 |
|  | 0.4348 | 0.3 | 0 | 0.7 | 3,537.4 | 400.1 | 0.33 | 767.21 |
|  | 0.4348 | 0.2 | 0 | 0.8 | 3,537.4 | 400.1 | 0.33 | 767.21 |
|  | 0.4348 | 0.1 | 0 | 0.9 | 3,537.4 | 400.1 | 0.33 | 767.21 |
|  | 0.4348 | 0 | 0 | 1 | 3,537.4 | 400.1 | 0.33 | 767.21 |
| SCXN-I | 0.4348 | 1 | 0 | 0 | 3,342.1 | 236.3 | 0.33 | 866.63 |
|  | 0.4348 | 0.9 | 0.1 | 0 | 3,342.1 | 236.3 | 0.33 | 866.63 |
|  | 0.4348 | 0.8 | 0.2 | 0 | 3,342.1 | 236.3 | 0.33 | 866.63 |
|  | 0.4348 | 0.7 | 0.3 | 0 | 3,342.1 | 236.3 | 0.33 | 866.63 |
|  | 0.4348 | 0.6 | 0.4 | 0 | 3,342.1 | 236.3 | 0.33 | 866.63 |
|  | 0.4348 | 0.5 | 0.5 | 0 | 3,342.1 | 236.3 | 0.33 | 866.63 |
|  | 0.4348 | 0.4 | 0.6 | 0 | 3,342.1 | 236.3 | 0.33 | 866.63 |
|  | 0.4348 | 0.3 | 0.7 | 0 | 3,342.1 | 236.3 | 0.33 | 866.63 |
|  | 0.4348 | 0.2 | 0.8 | 0 | 3,342.1 | 236.3 | 0.33 | 866.63 |
|  | 0.4348 | 0.1 | 0.9 | 0 | 3,342.1 | 236.3 | 0.33 | 866.63 |
|  | 0.4348 | 0 | 1 | 0 | 3,342.1 | 236.3 | 0.33 | 866.63 |
| SCPN-I | 0.4348 | 1 | 0 | 0 | 3,537.4 | 270.1 | 0.33 | 897.23 |
|  | 0.4348 | 0.9 | 0.1 | 0 | 3,517.9 | 270.1 | 0.33 | 890.79 |
|  | 0.4348 | 0.8 | 0.2 | 0 | 3,498.3 | 270.1 | 0.33 | 884.34 |
|  | 0.4348 | 0.7 | 0.3 | 0 | 3,478.8 | 270.1 | 0.33 | 877.90 |
|  | 0.4348 | 0.6 | 0.4 | 0 | 3,459.3 | 270.1 | 0.33 | 871.45 |
|  | 0.4348 | 0.5 | 0.5 | 0 | 3,439.7 | 270.1 | 0.33 | 865.00 |

Supporting Table S2. (continued)

| **Scenario** | **p_o_** | **p_w_** | **p_s_** | **p_c_** | ***E*(Y) (kg/ha)** | ***E*(C) ($/ha)** | ***E*(P) ($/kg)** | ***E*(R) ($/ha)** |
| --- | --- | --- | --- | --- | --- | --- | --- | --- |
|  | 0.4348 | 0.4 | 0.6 | 0 | 3,420.2 | 270.1 | 0.33 | 858.56 |
|  | 0.4348 | 0.3 | 0.7 | 0 | 3,400.7 | 270.1 | 0.33 | 852.11 |
|  | 0.4348 | 0.2 | 0.8 | 0 | 3,381.1 | 270.1 | 0.33 | 845.67 |
|  | 0.4348 | 0.1 | 0.9 | 0 | 3,361.6 | 270.1 | 0.33 | 839.22 |
|  | 0.4348 | 0 | 1 | 0 | 3,342.1 | 270.1 | 0.33 | 832.77 |
| SCPN-I | 0.4348 | 1 | 0 | 0 | 3,537.4 | 278.3 | 0.33 | 889.08 |
| (1B only) | 0.4348 | 0.9 | 0.1 | 0 | 3,537.4 | 278.3 | 0.33 | 889.08 |
|  | 0.4348 | 0.8 | 0.2 | 0 | 3,537.4 | 278.3 | 0.33 | 889.08 |
|  | 0.4348 | 0.7 | 0.3 | 0 | 3,537.4 | 278.3 | 0.33 | 889.08 |
|  | 0.4348 | 0.6 | 0.4 | 0 | 3,537.4 | 278.3 | 0.33 | 889.08 |
|  | 0.4348 | 0.5 | 0.5 | 0 | 3,537.4 | 278.3 | 0.33 | 889.08 |
|  | 0.4348 | 0.4 | 0.6 | 0 | 3,537.4 | 278.3 | 0.33 | 889.08 |
|  | 0.4348 | 0.3 | 0.7 | 0 | 3,537.4 | 278.3 | 0.33 | 889.08 |
|  | 0.4348 | 0.2 | 0.8 | 0 | 3,537.4 | 278.3 | 0.33 | 889.08 |
|  | 0.4348 | 0.1 | 0.9 | 0 | 3,537.4 | 278.3 | 0.33 | 889.08 |
|  | 0.4348 | 0 | 1 | 0 | 3,537.4 | 278.3 | 0.33 | 889.08 |
| SCPN-I | 0.4348 | 1 | 0 | 0 | 3,537.4 | 318.0 | 0.33 | 849.30 |
| (4C only) | 0.4348 | 0.9 | 0.1 | 0 | 3,537.4 | 318.0 | 0.33 | 849.30 |
|  | 0.4348 | 0.8 | 0.2 | 0 | 3,537.4 | 318.0 | 0.33 | 849.30 |
|  | 0.4348 | 0.7 | 0.3 | 0 | 3,537.4 | 318.0 | 0.33 | 849.30 |
|  | 0.4348 | 0.6 | 0.4 | 0 | 3,537.4 | 318.0 | 0.33 | 849.30 |
|  | 0.4348 | 0.5 | 0.5 | 0 | 3,537.4 | 318.0 | 0.33 | 849.30 |
|  | 0.4348 | 0.4 | 0.6 | 0 | 3,537.4 | 318.0 | 0.33 | 849.30 |
|  | 0.4348 | 0.3 | 0.7 | 0 | 3,537.4 | 318.0 | 0.33 | 849.30 |
|  | 0.4348 | 0.2 | 0.8 | 0 | 3,537.4 | 318.0 | 0.33 | 849.30 |
|  | 0.4348 | 0.1 | 0.9 | 0 | 3,537.4 | 318.0 | 0.33 | 849.30 |
|  | 0.4348 | 0 | 1 | 0 | 3,537.4 | 318.0 | 0.33 | 849.30 |

Supporting Table S2. (continued)

| **Scenario** | **p_o_** | **p_w_** | **p_s_** | **p_c_** | ***E*(Y) (kg/ha)** | ***E*(C) ($/ha)** | ***E*(P) ($/kg)** | ***E*(R) ($/ha)** |
| --- | --- | --- | --- | --- | --- | --- | --- | --- |
| SCPE-I | 0.4348 | 1 | 0 | 0 | 3,537.4 | 333.1 | 0.33 | 886.73 |
| (3A/1B) | 0.4348 | 0.9 | 0.1 | 0 | 3,537.4 | 333.1 | 0.33 | 881.48 |
|  | 0.4348 | 0.8 | 0.2 | 0 | 3,537.4 | 333.1 | 0.33 | 876.23 |
|  | 0.4348 | 0.7 | 0.3 | 0 | 3,537.4 | 333.1 | 0.33 | 870.98 |
|  | 0.4348 | 0.6 | 0.4 | 0 | 3,537.4 | 333.1 | 0.33 | 865.73 |
|  | 0.4348 | 0.5 | 0.5 | 0 | 3,537.4 | 333.1 | 0.33 | 860.48 |
|  | 0.4348 | 0.4 | 0.6 | 0 | 3,537.4 | 333.1 | 0.33 | 855.23 |
|  | 0.4348 | 0.3 | 0.7 | 0 | 3,537.4 | 333.1 | 0.33 | 849.98 |
|  | 0.4348 | 0.2 | 0.8 | 0 | 3,537.4 | 333.1 | 0.33 | 844.72 |
|  | 0.4348 | 0.1 | 0.9 | 0 | 3,537.4 | 333.1 | 0.33 | 839.47 |
|  | 0.4348 | 0 | 1 | 0 | 3,537.4 | 333.1 | 0.33 | 834.22 |
| SCPE-I | 0.4348 | 1 | 0 | 0 | 3,537.4 | 328.5 | 0.33 | 838.79 |
| (4C only) | 0.4348 | 0.9 | 0.1 | 0 | 3,537.4 | 328.5 | 0.33 | 838.79 |
|  | 0.4348 | 0.8 | 0.2 | 0 | 3,537.4 | 328.5 | 0.33 | 838.79 |
|  | 0.4348 | 0.7 | 0.3 | 0 | 3,537.4 | 328.5 | 0.33 | 838.79 |
|  | 0.4348 | 0.6 | 0.4 | 0 | 3,537.4 | 328.5 | 0.33 | 838.79 |
|  | 0.4348 | 0.5 | 0.5 | 0 | 3,537.4 | 328.5 | 0.33 | 838.79 |
|  | 0.4348 | 0.4 | 0.6 | 0 | 3,537.4 | 328.5 | 0.33 | 838.79 |
|  | 0.4348 | 0.3 | 0.7 | 0 | 3,537.4 | 328.5 | 0.33 | 838.79 |
|  | 0.4348 | 0.2 | 0.8 | 0 | 3,537.4 | 328.5 | 0.33 | 838.79 |
|  | 0.4348 | 0.1 | 0.9 | 0 | 3,537.4 | 328.5 | 0.33 | 838.79 |
|  | 0.4348 | 0 | 1 | 0 | 3,537.4 | 328.5 | 0.33 | 838.79 |
| SCPN-I | 0.4348 | 1 | 0 | 0 | 3,537.4 | 318.0 | 0.33 | 849.30 |
| (4C only) | 0.4348 | 0.9 | 0 | 0.1 | 3,537.4 | 318.0 | 0.33 | 849.30 |
|  | 0.4348 | 0.8 | 0 | 0.2 | 3,537.4 | 318.0 | 0.33 | 849.30 |
|  | 0.4348 | 0.7 | 0 | 0.3 | 3,537.4 | 318.0 | 0.33 | 849.30 |
|  | 0.4348 | 0.6 | 0 | 0.4 | 3,537.4 | 318.0 | 0.33 | 849.30 |
|  | 0.4348 | 0.5 | 0 | 0.5 | 3,537.4 | 318.0 | 0.33 | 849.30 |

Supporting Table S2. (continued)

| **Scenario** | **p_o_** | **p_w_** | **p_s_** | **p_c_** | ***E*(Y) (kg/ha)** | ***E*(C) ($/ha)** | ***E*(P) ($/kg)** | ***E*(R) ($/ha)** |
| --- | --- | --- | --- | --- | --- | --- | --- | --- |
|  | 0.4348 | 0.4 | 0 | 0.6 | 3,537.4 | 318.0 | 0.33 | 849.30 |
|  | 0.4348 | 0.3 | 0 | 0.7 | 3,537.4 | 318.0 | 0.33 | 849.30 |
|  | 0.4348 | 0.2 | 0 | 0.8 | 3,537.4 | 318.0 | 0.33 | 849.30 |
|  | 0.4348 | 0.1 | 0 | 0.9 | 3,537.4 | 318.0 | 0.33 | 849.30 |
|  | 0.4348 | 0 | 0 | 1 | 3,537.4 | 318.0 | 0.33 | 849.30 |
| SCPE-I | 0.4348 | 1 | 0 | 0 | 3,537.4 | 381.1 | 0.33 | 878.58 |
| (1B/4C) | 0.4348 | 0.9 | 0 | 0.1 | 3,537.4 | 381.1 | 0.33 | 869.35 |
|  | 0.4348 | 0.8 | 0 | 0.2 | 3,537.4 | 381.1 | 0.33 | 860.12 |
|  | 0.4348 | 0.7 | 0 | 0.3 | 3,537.4 | 381.1 | 0.33 | 850.89 |
|  | 0.4348 | 0.6 | 0 | 0.4 | 3,537.4 | 381.1 | 0.33 | 841.66 |
|  | 0.4348 | 0.5 | 0 | 0.5 | 3,537.4 | 381.1 | 0.33 | 832.43 |
|  | 0.4348 | 0.4 | 0 | 0.6 | 3,537.4 | 381.1 | 0.33 | 823.20 |
|  | 0.4348 | 0.3 | 0 | 0.7 | 3,537.4 | 381.1 | 0.33 | 813.97 |
|  | 0.4348 | 0.2 | 0 | 0.8 | 3,537.4 | 381.1 | 0.33 | 804.74 |
|  | 0.4348 | 0.1 | 0 | 0.9 | 3,537.4 | 381.1 | 0.33 | 795.51 |
|  | 0.4348 | 0 | 0 | 1 | 3,537.4 | 381.1 | 0.33 | 786.29 |
| SCTM-I | 0.4348 | 1 | 0 | 0 | 3,537.4 | 396.1 | 0.33 | 823.72 |
| (3A/1B) | 0.4348 | 0.9 | 0.1 | 0 | 3,537.4 | 396.1 | 0.33 | 818.47 |
|  | 0.4348 | 0.8 | 0.2 | 0 | 3,537.4 | 396.1 | 0.33 | 813.22 |
|  | 0.4348 | 0.7 | 0.3 | 0 | 3,537.4 | 396.1 | 0.33 | 807.97 |
|  | 0.4348 | 0.6 | 0.4 | 0 | 3,537.4 | 396.1 | 0.33 | 802.72 |
|  | 0.4348 | 0.5 | 0.5 | 0 | 3,537.4 | 396.1 | 0.33 | 797.47 |
|  | 0.4348 | 0.4 | 0.6 | 0 | 3,537.4 | 396.1 | 0.33 | 792.22 |
|  | 0.4348 | 0.3 | 0.7 | 0 | 3,537.4 | 396.1 | 0.33 | 786.96 |
|  | 0.4348 | 0.2 | 0.8 | 0 | 3,537.4 | 396.1 | 0.33 | 781.71 |
|  | 0.4348 | 0.1 | 0.9 | 0 | 3,537.4 | 396.1 | 0.33 | 776.46 |
|  | 0.4348 | 0 | 1 | 0 | 3,537.4 | 396.1 | 0.33 | 771.21 |

Supporting Table S2. (continued)

| **Scenario** | **p_o_** | **p_w_** | **p_s_** | **p_c_** | ***E*(Y) (kg/ha)** | ***E*(C) ($/ha)** | ***E*(P) ($/kg)** | ***E*(R) ($/ha)** |
| --- | --- | --- | --- | --- | --- | --- | --- | --- |
| SCTM-I | 0.4348 | 1 | 0 | 0 | 3,537.4 | 351.8 | 0.33 | 815.57 |
| (1B only) | 0.4348 | 0.9 | 0.1 | 0 | 3,537.4 | 351.8 | 0.33 | 815.57 |
|  | 0.4348 | 0.8 | 0.2 | 0 | 3,537.4 | 351.8 | 0.33 | 815.57 |
|  | 0.4348 | 0.7 | 0.3 | 0 | 3,537.4 | 351.8 | 0.33 | 815.57 |
|  | 0.4348 | 0.6 | 0.4 | 0 | 3,537.4 | 351.8 | 0.33 | 815.57 |
|  | 0.4348 | 0.5 | 0.5 | 0 | 3,537.4 | 351.8 | 0.33 | 815.57 |
|  | 0.4348 | 0.4 | 0.6 | 0 | 3,537.4 | 351.8 | 0.33 | 815.57 |
|  | 0.4348 | 0.3 | 0.7 | 0 | 3,537.4 | 351.8 | 0.33 | 815.57 |
|  | 0.4348 | 0.2 | 0.8 | 0 | 3,537.4 | 351.8 | 0.33 | 815.57 |
|  | 0.4348 | 0.1 | 0.9 | 0 | 3,537.4 | 351.8 | 0.33 | 815.57 |
|  | 0.4348 | 0 | 1 | 0 | 3,537.4 | 351.8 | 0.33 | 815.57 |
| SCTM-I | 0.4348 | 1 | 0 | 0 | 3,537.4 | 391.6 | 0.33 | 775.78 |
| (4C only) | 0.4348 | 0.9 | 0.1 | 0 | 3,537.4 | 391.6 | 0.33 | 775.78 |
|  | 0.4348 | 0.8 | 0.2 | 0 | 3,537.4 | 391.6 | 0.33 | 775.78 |
|  | 0.4348 | 0.7 | 0.3 | 0 | 3,537.4 | 391.6 | 0.33 | 775.78 |
|  | 0.4348 | 0.6 | 0.4 | 0 | 3,537.4 | 391.6 | 0.33 | 775.78 |
|  | 0.4348 | 0.5 | 0.5 | 0 | 3,537.4 | 391.6 | 0.33 | 775.78 |
|  | 0.4348 | 0.4 | 0.6 | 0 | 3,537.4 | 391.6 | 0.33 | 775.78 |
|  | 0.4348 | 0.3 | 0.7 | 0 | 3,537.4 | 391.6 | 0.33 | 775.78 |
|  | 0.4348 | 0.2 | 0.8 | 0 | 3,537.4 | 391.6 | 0.33 | 775.78 |
|  | 0.4348 | 0.1 | 0.9 | 0 | 3,537.4 | 391.6 | 0.33 | 775.78 |
|  | 0.4348 | 0 | 1 | 0 | 3,537.4 | 391.6 | 0.33 | 775.78 |
| SCTM-I | 0.4348 | 1 | 0 | 0 | 3,537.4 | 444.1 | 0.33 | 815.57 |
| (1B/4C) | 0.4348 | 0.9 | 0 | 0.1 | 3,537.4 | 444.1 | 0.33 | 806.34 |
|  | 0.4348 | 0.8 | 0 | 0.2 | 3,537.4 | 444.1 | 0.33 | 797.11 |
|  | 0.4348 | 0.7 | 0 | 0.3 | 3,537.4 | 444.1 | 0.33 | 787.88 |
|  | 0.4348 | 0.6 | 0 | 0.4 | 3,537.4 | 444.1 | 0.33 | 778.65 |
|  | 0.4348 | 0.5 | 0 | 0.5 | 3,537.4 | 444.1 | 0.33 | 769.42 |

Supporting Table S2. (continued)

| **Scenario** | **p_o_** | **p_w_** | **p_s_** | **p_c_** | ***E*(Y) (kg/ha)** | ***E*(C) ($/ha)** | ***E*(P) ($/kg)** | ***E*(R) ($/ha)** |
| --- | --- | --- | --- | --- | --- | --- | --- | --- |
|  | 0.4348 | 0.4 | 0 | 0.6 | 3,537.4 | 444.1 | 0.33 | 760.19 |
|  | 0.4348 | 0.3 | 0 | 0.7 | 3,537.4 | 444.1 | 0.33 | 750.96 |
|  | 0.4348 | 0.2 | 0 | 0.8 | 3,537.4 | 444.1 | 0.33 | 741.73 |
|  | 0.4348 | 0.1 | 0 | 0.9 | 3,537.4 | 444.1 | 0.33 | 732.50 |
|  | 0.4348 | 0 | 0 | 1 | 3,537.4 | 444.1 | 0.33 | 723.27 |
| SCTM-I | 0.4348 | 1 | 0 | 0 | 3,537.4 | 391.6 | 0.33 | 775.78 |
| (4C only) | 0.4348 | 0.9 | 0 | 0.1 | 3,537.4 | 391.6 | 0.33 | 775.78 |
|  | 0.4348 | 0.8 | 0 | 0.2 | 3,537.4 | 391.6 | 0.33 | 775.78 |
|  | 0.4348 | 0.7 | 0 | 0.3 | 3,537.4 | 391.6 | 0.33 | 775.78 |
|  | 0.4348 | 0.6 | 0 | 0.4 | 3,537.4 | 391.6 | 0.33 | 775.78 |
|  | 0.4348 | 0.5 | 0 | 0.5 | 3,537.4 | 391.6 | 0.33 | 775.78 |
|  | 0.4348 | 0.4 | 0 | 0.6 | 3,537.4 | 391.6 | 0.33 | 775.78 |
|  | 0.4348 | 0.3 | 0 | 0.7 | 3,537.4 | 391.6 | 0.33 | 775.78 |
|  | 0.4348 | 0.2 | 0 | 0.8 | 3,537.4 | 391.6 | 0.33 | 775.78 |
|  | 0.4348 | 0.1 | 0 | 0.9 | 3,537.4 | 391.6 | 0.33 | 775.78 |
|  | 0.4348 | 0 | 0 | 1 | 3,537.4 | 391.6 | 0.33 | 775.78 |
| RCXN-I | 0.4348 | 1 | 0 | 0 | 3,537.4 | 233.0 | 0.33 | 934.37 |
|  | 0.4348 | 0.9 | 0.1 | 0 | 3,537.4 | 233.0 | 0.33 | 934.37 |
|  | 0.4348 | 0.8 | 0.2 | 0 | 3,537.4 | 233.0 | 0.33 | 934.37 |
|  | 0.4348 | 0.7 | 0.3 | 0 | 3,537.4 | 233.0 | 0.33 | 934.37 |
|  | 0.4348 | 0.6 | 0.4 | 0 | 3,537.4 | 233.0 | 0.33 | 934.37 |
|  | 0.4348 | 0.5 | 0.5 | 0 | 3,537.4 | 233.0 | 0.33 | 934.37 |
|  | 0.4348 | 0.4 | 0.6 | 0 | 3,537.4 | 233.0 | 0.33 | 934.37 |
|  | 0.4348 | 0.3 | 0.7 | 0 | 3,537.4 | 233.0 | 0.33 | 934.37 |
|  | 0.4348 | 0.2 | 0.8 | 0 | 3,537.4 | 233.0 | 0.33 | 934.37 |
|  | 0.4348 | 0.1 | 0.9 | 0 | 3,537.4 | 233.0 | 0.33 | 934.37 |
|  | 0.4348 | 0 | 1 | 0 | 3,537.4 | 233.0 | 0.33 | 934.37 |
| RCXN-I | 0.4348 | 1 | 0 | 0 | 3,537.4 | 233.0 | 0.33 | 934.37 |

Supporting Table S2. (continued)

| **Scenario** | **p_o_** | **p_w_** | **p_s_** | **p_c_** | ***E*(Y) (kg/ha)** | ***E*(C) ($/ha)** | ***E*(P) ($/kg)** | ***E*(R) ($/ha)** |
| --- | --- | --- | --- | --- | --- | --- | --- | --- |
|  | 0.4348 | 0.9 | 0 | 0.1 | 3,537.4 | 233.0 | 0.33 | 934.37 |
|  | 0.4348 | 0.8 | 0 | 0.2 | 3,537.4 | 233.0 | 0.33 | 934.37 |
|  | 0.4348 | 0.7 | 0 | 0.3 | 3,537.4 | 233.0 | 0.33 | 934.37 |
|  | 0.4348 | 0.6 | 0 | 0.4 | 3,537.4 | 233.0 | 0.33 | 934.37 |
|  | 0.4348 | 0.5 | 0 | 0.5 | 3,537.4 | 233.0 | 0.33 | 934.37 |
|  | 0.4348 | 0.4 | 0 | 0.6 | 3,537.4 | 233.0 | 0.33 | 934.37 |
|  | 0.4348 | 0.3 | 0 | 0.7 | 3,537.4 | 233.0 | 0.33 | 934.37 |
|  | 0.4348 | 0.2 | 0 | 0.8 | 3,537.4 | 233.0 | 0.33 | 934.37 |
|  | 0.4348 | 0.1 | 0 | 0.9 | 3,537.4 | 233.0 | 0.33 | 934.37 |
|  | 0.4348 | 0 | 0 | 1 | 3,537.4 | 233.0 | 0.33 | 934.37 |
| RCXM-I | 0.4348 | 1 | 0 | 0 | 3,537.4 | 296.0 | 0.33 | 871.36 |
|  | 0.4348 | 0.9 | 0.1 | 0 | 3,537.4 | 296.0 | 0.33 | 871.36 |
|  | 0.4348 | 0.8 | 0.2 | 0 | 3,537.4 | 296.0 | 0.33 | 871.36 |
|  | 0.4348 | 0.7 | 0.3 | 0 | 3,537.4 | 296.0 | 0.33 | 871.36 |
|  | 0.4348 | 0.6 | 0.4 | 0 | 3,537.4 | 296.0 | 0.33 | 871.36 |
|  | 0.4348 | 0.5 | 0.5 | 0 | 3,537.4 | 296.0 | 0.33 | 871.36 |
|  | 0.4348 | 0.4 | 0.6 | 0 | 3,537.4 | 296.0 | 0.33 | 871.36 |
|  | 0.4348 | 0.3 | 0.7 | 0 | 3,537.4 | 296.0 | 0.33 | 871.36 |
|  | 0.4348 | 0.2 | 0.8 | 0 | 3,537.4 | 296.0 | 0.33 | 871.36 |
|  | 0.4348 | 0.1 | 0.9 | 0 | 3,537.4 | 296.0 | 0.33 | 871.36 |
|  | 0.4348 | 0 | 1 | 0 | 3,537.4 | 296.0 | 0.33 | 871.36 |
| RCXM-I | 0.4348 | 1 | 0 | 0 | 3,537.4 | 296.0 | 0.33 | 871.36 |
|  | 0.4348 | 0.9 | 0 | 0.1 | 3,537.4 | 296.0 | 0.33 | 871.36 |
|  | 0.4348 | 0.8 | 0 | 0.2 | 3,537.4 | 296.0 | 0.33 | 871.36 |
|  | 0.4348 | 0.7 | 0 | 0.3 | 3,537.4 | 296.0 | 0.33 | 871.36 |
|  | 0.4348 | 0.6 | 0 | 0.4 | 3,537.4 | 296.0 | 0.33 | 871.36 |
|  | 0.4348 | 0.5 | 0 | 0.5 | 3,537.4 | 296.0 | 0.33 | 871.36 |
|  | 0.4348 | 0.4 | 0 | 0.6 | 3,537.4 | 296.0 | 0.33 | 871.36 |

Supporting Table S2. (continued)

| **Scenario** | **p_o_** | **p_w_** | **p_s_** | **p_c_** | ***E*(Y) (kg/ha)** | ***E*(C) ($/ha)** | ***E*(P) ($/kg)** | ***E*(R) ($/ha)** |
| --- | --- | --- | --- | --- | --- | --- | --- | --- |
|  | 0.4348 | 0.3 | 0 | 0.7 | 3,537.4 | 296.0 | 0.33 | 871.36 |
|  | 0.4348 | 0.2 | 0 | 0.8 | 3,537.4 | 296.0 | 0.33 | 871.36 |
|  | 0.4348 | 0.1 | 0 | 0.9 | 3,537.4 | 296.0 | 0.33 | 871.36 |
|  | 0.4348 | 0 | 0 | 1 | 3,537.4 | 296.0 | 0.33 | 871.36 |

1. Refer to Materials and Methods section of the manuscript for a description of scenario abbreviations and terms. [↑](#footnote-ref-1)
2. The probability of an outbreak occurring (p_o_). Within an outbreak, aphids could be wild-type (p_w_), pyrethroid-resistant (p_s_), or cross-resistant to organophosphates (p_c_). [↑](#footnote-ref-2)
3. The expected yield calculated using Equation 1; also in Supporting Table S1. [↑](#footnote-ref-3)
4. The variable costs associated with each scenario: costs for herbicides, seed, insecticides + application, and scouting. [↑](#footnote-ref-4)
5. The calendar year market price of soybean in Iowa in 2018. [↑](#footnote-ref-5)
6. Expected net revenue calculated using Equation 2 and these parameter values. [↑](#footnote-ref-6)
